# Comparative genomic analyses of nickel, cobalt and vitamin B12 utilization

**DOI:** 10.1186/1471-2164-10-78

**Published:** 2009-02-10

**Authors:** Yan Zhang, Dmitry A Rodionov, Mikhail S Gelfand, Vadim N Gladyshev

**Affiliations:** 1 Department of Biochemistry and Redox Biology Center, University of Nebraska, Lincoln, NE 68588-0664, USA; 2 Institute for Information Transmission Problems (the Kharkevich Institute), Russian Academy of Sciences, Moscow, 127994, Russia; 3 Burnham Institute for Medical Research, La Jolla, CA 92037, USA; 4 Faculty of Bioengineering and Bioinformatics, Moscow State University, Moscow, 119992, Russia

## Abstract

**Background:**

Nickel (Ni) and cobalt (Co) are trace elements required for a variety of biological processes. Ni is directly coordinated by proteins, whereas Co is mainly used as a component of vitamin B_12_. Although a number of Ni and Co-dependent enzymes have been characterized, systematic evolutionary analyses of utilization of these metals are limited.

**Results:**

We carried out comparative genomic analyses to examine occurrence and evolutionary dynamics of the use of Ni and Co at the level of (i) transport systems, and (ii) metalloproteomes. Our data show that both metals are widely used in bacteria and archaea. Cbi/NikMNQO is the most common prokaryotic Ni/Co transporter, while Ni-dependent urease and Ni-Fe hydrogenase, and B_12_-dependent methionine synthase (MetH), ribonucleotide reductase and methylmalonyl-CoA mutase are the most widespread metalloproteins for Ni and Co, respectively. Occurrence of other metalloenzymes showed a mosaic distribution and a new B_12_-dependent protein family was predicted. *Deltaproteobacteria *and *Methanosarcina *generally have larger Ni- and Co-dependent proteomes. On the other hand, utilization of these two metals is limited in eukaryotes, and very few of these organisms utilize both of them. The Ni-utilizing eukaryotes are mostly fungi (except saccharomycotina) and plants, whereas most B_12_-utilizing organisms are animals. The NiCoT transporter family is the most widespread eukaryotic Ni transporter, and eukaryotic urease and MetH are the most common Ni- and B_12_-dependent enzymes, respectively. Finally, investigation of environmental and other conditions and identity of organisms that show dependence on Ni or Co revealed that host-associated organisms (particularly obligate intracellular parasites and endosymbionts) have a tendency for loss of Ni/Co utilization.

**Conclusion:**

Our data provide information on the evolutionary dynamics of Ni and Co utilization and highlight widespread use of these metals in the three domains of life, yet only a limited number of user proteins.

## Background

Life is dependent on a number of chemical elements. Besides common elements, several trace elements are utilized, including certain metals and metalloids. Because these elements play important roles in cellular metabolism, efficient mechanisms of uptake, storage and utilization are required for many of them. Among biometals, nickel (Ni) and cobalt (Co) are utilized at particularly low levels but play important roles in several biological systems.

Ni is an essential component of several metalloenzymes involved in energy and nitrogen metabolism [1,2]. In prokaryotes, the major Ni-binding enzymes include urease, Ni-Fe hydrogenase, carbon monoxide dehydrogenase (Ni-CODH), acetyl-coenzyme A decarbonylase/synthase ([4Fe-4S]-Ni-Ni CODH/ACS), superoxide dismutase SodN, methyl-coenzyme M reductase (MCR), glyoxalase I (GlxI, binds Ni in *Escherichia coli*, *Pseudomonas aeruginosa *and *Neisseria meningitidis*, but zinc in *P. putida*, human and yeast) [3-6], a putative cis-trans isomerase in *E. coli *[7] and several other proteins [2]. In eukaryotes, urease is the only characterized Ni-dependent enzyme [8]. Additional candidate Ni-containing proteins or compounds have also been described in different organisms including humans [9].

Co is mainly found in the corrin ring of vitamin B_12 _(also known as cobalamin), a group of closely related polypyrrole compounds such as cyanocobalamin, methylcobalamin and deoxyadenosyl cobalamin [10-12]. The biochemistry of B_12 _in enzymes is well characterized [10-12]. Vitamin B_12 _is a complex organometallic cofactor and is mainly present in three classes of enzymes in prokaryotes (classified based on different chemical features of the cofactor): adenosylcobalamin-dependent isomerase, methylcobalamin-dependent methyltransferase, and B_12_-dependent reductive dehalogenase [12]. These classes can be further divided into subclasses based on sequence similarity and reactions they catalyze, including methylmalonyl-CoA mutase (MCM), isobutyryl-CoA mutase (ICM), B_12_-dependent mutase MeaA (with sequence similarity to MCM and ICM), glutamate mutase (GM), methyleneglutarate mutase (MGM), D-lysine 5,6-aminomutase (5,6-LAM), B_12_-dependent ribonucleotide reductase (RNR II), diol dehydratase (DDH), ethanolamine ammonia lyase (EAL), B_12_-dependent methionine synthase (MetH), a variety of B_12_-dependent methyltransferases (such as Mta, Mtm, Mtb, Mtt, Mts, Mtv and Mtr) and reductive dehalogenases CprA and PceA [12-18]. Whereas many prokaryotes synthesize B_12 _via aerobic or anaerobic biosynthetic pathways [11], other organisms, which lack the ability to synthesize B_12_, are dependent on vitamin uptake from the environment. In eukaryotes, only three B_12_-dependent enzymes, MetH, MCM and RNR II, have been identified [19,20], and all are dependent on externally supplied vitamin B_12_. Besides, a few proteins containing non-corrin Co were reported, such as methionine aminopeptidase from *Salmonella typhimurium*, prolidase from *Pyrococcus furiosus *and nitrile hydratase from *Rhodococcus rhodochrous *[10]. However, most of these proteins are not strictly Co-specific and may also use other metals (such as iron, zinc and manganese) in place of Co [10,21,22]. Among them, only nitrile hydratase (NHase) was previously suggested to have different active site motifs for cobalt- and iron-binding forms [23,24].

Biosynthesis of Ni and Co enzymes is dependent on high-affinity uptake of metal ions from natural environments. In microorganisms, Ni and Co uptake is mediated by ATP-binding cassette (ABC) systems and several secondary transporters [25,26]. The well-studied ABC-type Ni transporter system, NikABCDE, belongs to a large family of ABC transporters (peptide/nickel transporter family). It is composed of a periplasmic binding protein (NikA), two integral membrane proteins (NikB and NikC) and two ABC proteins (NikD and NikE, [27]). The expression of *nikABCDE *is negatively regulated by the NikR repressor [28]. Distantly related Ni ABC transporters were also identified in the *Yersinia *species (YntABCDE, [29]). An additional system, Cbi/NikMNQO, is often encoded next to the B_12 _biosynthesis or urease genes in bacterial genomes [30-33]. It was shown to mediate Co and Ni uptake, respectively [30,31].

Secondary Ni/Co transporters include: (a) NiCoT (also designated HoxN, HupN, NicT, NixA or NhlF in different organisms), a family of prokaryotic and fungal membrane proteins with an eight-transmembrane-segment structure [34-36], (b) UreH [26] and (c) HupE/UreJ [26,37]. NiCoTs are widespread among bacteria and found in several thermoacidophilic archaea and certain fungi including *Schizosaccharomyces pombe *and *Neurospora crassa *[26,36,38]. Subtypes of various NiCoTs have different ion preferences ranging from strict selectivity for Ni to unbiased transport of both ions to strong preference for Co. In many cases, the preference for a particular metal correlated with the genomic location of NiCoT genes, which are adjacent to genes for Ni or Co (or B_12 _biosynthesis) enzymes [31,34-36]. The other two families (UreH and HupE/UreJ) are putative secondary transporters, and certain members of these families have recently been shown to mediate Ni transport [26,37,39]. Homologs of UreH also occur in plants [26]. Recently, several new types of candidate cobalt transporters were predicted, including CbtAB, CbtC, CbtD, CbtE, CbtF, CbtG and CbtX [31,40]. The distribution of these candidates is limited. In eukaryotes, a subfamily of cation-efflux family members (TgMTP1) was found to account for the enhanced ability of Ni hyperaccumulation in higher plants [41,42]. Although no Co-specific transport system was reported in eukaryotes, some suppressors of Co toxicity, such as COT1 and GRR1 in *Saccharomyces cerevisiae*, were characterized, which have a role in decreasing the cytoplasmic concentration of metal ions (including cobalt and zinc). They were proposed to play an important role in metal homeostasis [10].

Vitamin B_12 _uptake is essential for B_12_-utilizing organisms, which lack the ability to synthesize the coenzyme *de novo*, and the only known transport system for B_12 _in prokaryotes is BtuFCD [43]. Since this ABC transport system belongs to the same family as the ABC systems involved in the uptake of iron, siderophores and heme [44], it is difficult to distinguish the B_12_-specific transporters from other homologous transporters, especially in distantly related species. In mammals, B_12 _delivery from food to tissues involves at least three successive transport proteins and their cell-surface receptors: haptocorrin in saliva, intrinsic factor in the proximal ileum and the transcobalamin II in vascular endothelium [45]. Transcobalamin-B_12 _is then released to the plasma and enters cells by endocytosis via certain receptors [46]. However, the mechanism of B_12 _uptake in other eukaryotes, such as *Chlamydomonas reinhardtii *and nematodes, is unclear.

While a variety of metal transport systems and metalloproteomes have been characterized, the full details of utilization of Ni and Co/B_12 _are not clear. Comprehensive analyses of both transporters and proteins that bind these metals are essential for better understanding of their homeostasis and its changes during evolution. Recently, a comparative and functional genomic analysis of prokaryotic Ni and Co transporters in 200 microbial genomes showed a mosaic utilization of both metals [47]. A separate analysis of B_12 _metabolism and regulation provided information on B_12 _utilization in prokaryotes [31].

In this report, we used comparative genomics approaches to better understand Ni and Co uptake in both prokaryotes and eukaryotes, and consequently utilization of these trace elements. Considering that members of most non-corrin Co-binding proteins may bind other metal cofactors instead of Co, we only focused on the utilization of the corrin form of Co (vitamin B_12_), whose utilization could be predicted on the basis of B_12 _biosynthesis pathway and B_12_-dependent protein families. Over 740 organisms in all three domains of life were examined. Our results show a widespread utilization of both metals in prokaryotes and their limited use in eukaryotes, and reveal that utilization of Ni and Co may be influenced by environmental or other factors. These studies also provide insights into understanding the evolution of metal utilization traits and metalloenzymes.

## Results

### Occurrence of nickel and cobalt utilization in prokaryotes and eukaryotes

Analysis of prokaryotic genomes revealed a wide distribution of genes encoding Ni and Co transporters as well as Ni- and Co-dependent proteins [see Additional files 1 and 2]. Table 1 shows the general distribution of both utilization traits in the three domains of life. This analysis was carried out by detecting known metalloproteins, metal transporters and cofactor biosynthesis pathways, and where possible, calls were based on multiple evidences. It should be noted, however, that these approaches may occasionally be insufficient to assign a function with complete confidence. For example, it cannot be excluded that some genes said to be associated with Ni or Co utilization may prove to have a different metal specificity or may not be functional. Therefore, our analysis is consistent with the current knowledge of Ni and Co pathways.

**Table 1:** General distribution of Ni and Co utilization in the three domains of life

		**Archaea**	**Bacteria**	**Eukarya**	**Total**
**Ni-utilizing organisms**		**39**	**319**	**51**	**409**
**Ni User (+)**	Ni Transporter* (+)	21	166	49	236
	**Ni Transporter (-)** **& Unassigned transporter (+)**	11	81	-	92
	**Ni Transporter (-)** **& Unassigned transporter (-)**	7	62	2	71

**Ni User (-)**	**Ni Transporter (+)**	-	10	-	10
**Co-utilizing organisms**		**45**	**410**	**49**	**504**
**B**_12 _**biosynthesis pathway (+)**	**Co Transporter (+)**	15	180	-	195
	**Co Transporter (-)** **& Unassigned transporter (+)**	10	16	-	26
	**Co Transporter (-)** **& Unassigned transporter (-)**	9	13	-	22
**B**_12_** biosynthesis pathway (-)**	**Co Transporter (+)**	-	-	-	-
**Other (using external B**_12_**)**		11	201	49	261

**Organisms that use both Ni and Co**		**38**	**287**	**9**	**335**

**Organisms that use neither Ni nor Co**		**1**	**98**	**69**	**168**

*: Ni transporter: Ni-specific transporter;

Co transporter: Co-specific transporter;

Unassigned transporter: close homologs of Ni/Co transporter families with unassigned function.

Among bacteria, 319 Ni-utilizing and 410 Co-utilizing organisms (59.1% and 75.9% of sequenced bacterial species, respectively) were identified, including 287 organisms (53.1%) that utilized both metals. In contrast, 98 organisms (18.1%) had neither Ni/Co transporters nor corresponding metalloenzymes and appeared to lack the ability to use either of the two trace elements. Only half of Co-utilizing organisms (209 out of 410) possessed the B_12 _biosynthetic pathway. The other half likely acquires external B_12 _via the vitamin uptake systems. Investigation of the occurrence of homologs of the BtuFCD transport system in these B_12_-uptaking organisms showed that more than 90% of them had BtuFCD homologs, implying that essentially all of these organisms may use a BtuFCD system for B_12 _uptake [see Additional file 1]. The remaining 10% B_12_-uptaking organisms, such as *Nitrosomonas europaea *and *Xanthomonas axonopodis*, appeared to lack BtuFCD transporters, suggesting the presence of additional B_12 _transport systems in these organisms. A small number of organisms which had either Ni-dependent proteins (but lacked both Ni transporters and transporters with unassigned function) or Ni transporters (but lacked known Ni-dependent proteins) were found among bacteria (62 and 10 organisms, respectively, Table 1). A similar situation was also observed in 13 B_12_-synthesizing species that lacked both Co transporters and transporters with unassigned function. Therefore, our data suggest that dual-function Ni/Co transporters (i.e., some predicted Ni-specific transporters may also be involved in Co uptake), additional Ni- and Co-specific transporters, multifunctional metal transporters (e.g., magnesium/nickel/cobalt transport system) and/or novel metalloproteins may be present in a small number of analyzed organisms. Alternatively, metal acquisition might occur nonspecifically in some of these organisms using cation influx systems.

**Figure 1:**
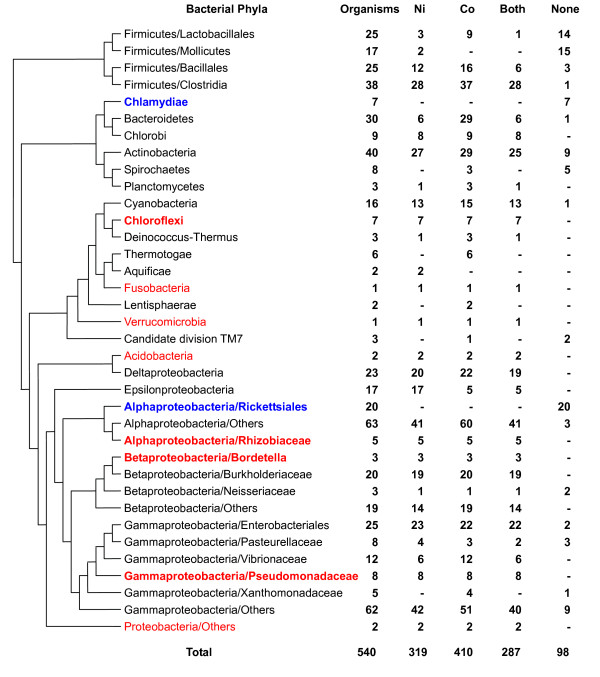
**Occurrence of nickel and cobalt utilization traits in bacteria**. The tree is based on a highly resolved phylogenetic tree of life (see Methods). We simplified the complete tree and only show bacterial branches. Phyla in which none of the organisms use Ni or Co are shown in blue (if containing at least 3 organisms, shown in bold). Phyla in which all organisms use both Ni and Co are shown in red (if containing at least 3 organisms, shown in bold).

Except for phyla represented by few sequenced organisms (<3), Ni and Co utilization traits were detected in nearly all bacterial phyla (Fig. 1). Neither Ni- nor Co-utilizing organisms were found among the *Chlamydiae *and *Alphaproteobacteria/Rickettsiales*. Essentially all organisms in the two phyla are obligate intracellular parasites and have small genome size (<1.5 Mbp). In addition, most organisms in the *Firmicutes/Mollicutes *(88.2%) and *Spirochaetes *(62.5%), which are extracellular parasites with small genomes, also lost the ability to use both metals. Thus, it appears that parasitic lifestyle may result in the loss of utilization of both metals. Co utilization appeared to be more widely distributed than that of Ni. It is present in 90% Ni-utilizing organisms and in some phyla, such as the *Spirochaetes *and *Thermotogae*, which lack Ni utilization. However, the fact that Ni utilization is found in all sequenced *Epsilonproteobacteria*, which rarely use Co, suggests a mostly independent relationship between the two metal utilization traits. Nevertheless, significant overlap between the two traits observed in bacteria suggests that they may be related in some way, for example, common or similar transporter systems may be involved.

**Figure 2:**
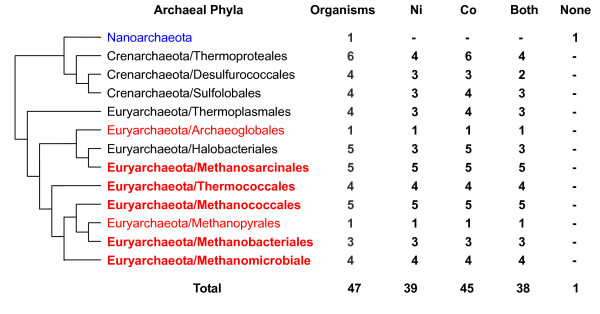
**Occurrence of nickel and cobalt utilization traits in archaea**. Phyla in which none of the organisms use Ni or Co are shown in blue (if containing at least 3 organisms, shown in bold). Phyla in which all organisms use both Ni and Co are shown in red (if containing at least 3 organisms, shown in bold).

Similar but even wider Ni/Co utilization was observed in sequenced archaea (Fig. 2 and [Additional file 2]). 45 and 39 archaeal species were found to utilize Co and Ni, respectively. A total of 38 organisms use both metals, including all 18 sequenced methanogenic archaea. Approximately 75% of Co-utilizing archaea possessed the B_12 _biosynthetic pathway (Table 1). Overall, it appears that utilization of both Ni and Co represent ancient traits which have been and remain common to most prokaryotes.

**Figure 3:**
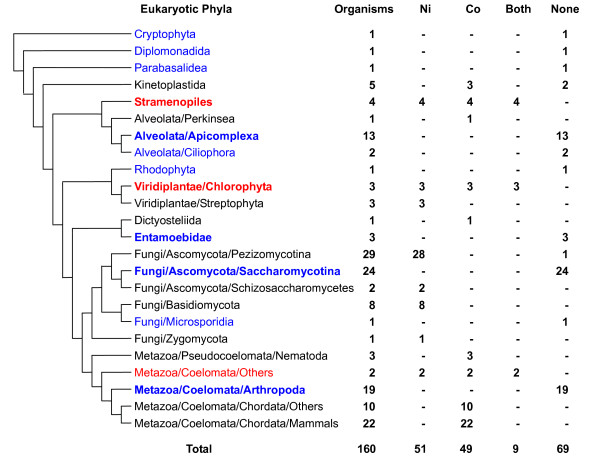
**Occurrence of nickel and cobalt utilization traits in eukaryotes**. Phyla in which none of the organisms use Ni or Co are shown in blue (if containing at least 3 organisms, shown in bold). Phyla in which all organisms use both Ni and Co are shown in red (if containing at least 3 organisms, shown in bold).

In contrast to prokaryotes, only 51 Ni-utilizing and 49 B_12_-utilizing organisms were identified in eukaryotes (31.9% and 30.6% of sequenced eukaryotic genomes, respectively). Among them, 9 organisms (belonging to the *Stramenopiles, Viridiplantae/Chlorophyta *and *Metazoa/Coelomata/Others*) use both trace elements (Fig. 3 and [Additional file 3]). On the other hand, almost half of analyzed eukaryotic organisms appeared to lack the ability to use either Ni or B_12_, including insects (*Metazoa/Coelomata/Arthropoda*), saccharomycotina and most unicellular parasites. The fact that no organism contained orphan Ni transporter and that more than 96% of Ni-utilizing eukaryotes possessed both known Ni transporters and urease (the only known Ni-dependent enzyme in eukaryotes) strongly suggested excellent correspondence between the occurrence of the Ni uptake system and Ni-dependent proteins in eukaryotes. Although the mechanism of B_12 _uptake is unclear in eukaryotes excluding mammals, we could examine B_12 _utilization by analyzing the occurrence of B_12_-dependent enzymes. It is interesting that most Ni-utilizing eukaryotes were fungi (including the *Ascomycota/Pezizomycotina*, *Ascomycota/Schizosaccharomycetes *and *Basidiomycota *subdivisions) and plants, and that most B_12_-utilizing organisms were animals (except insects) which lack the ability to use Ni (Fig. 3). The data suggest that the majority of lower eukaryotes lost the Co (or more precisely, B_12_) utilization trait whereas higher eukaryotes lost the Ni utilization trait. Although less likely, an alternative hypothesis is that the Co utilization trait was independently acquired by some ancient eukaryotes, for example, the ancestor of all animals, and then lost by certain groups such as arthropoda.

### Distribution of Ni and Co transporters in prokaryotes

We analyzed all well-characterized Ni/Co transport systems in prokaryotes [26,31,40,47]. Members of these transporter families in sequenced genomes were identified by homology searches and the function of each protein was predicted based on genome context (see Methods). Orthologs of these transporters showed a mosaic distribution in bacteria. A summary of the distribution of these Ni/Co transporters in bacteria is shown in Table 2. Considering that many transporters do not have clear substrate preference (either Ni or Co or both), our analyses focused on predicted Ni- or Co-specific transporters. Although some transporters with unassigned function were clustered with multiple predicted Ni- or Co-specific transporters in phylogenetic trees, we considered them as being of unclear function.

**Table 2:** Distribution of Ni/Co transporters in bacteria

**Phylum**	**Total organisms**	**CbiMNQO** /**NikMNQO** /**NikKMLQO**			**NikABCDE**			**NiCoT**			**UreH**			**HupE/UreJ**			Other predicted Co transporters**
			
		N*	**C**	**U**	**N**	**C**	**U**	**N**	**C**	**U**	**N**	**C**	**U**	**N**	**C**	**U**	
Firmicutes/Lactobacillales	25	1	2	2	2	-	-	-	-	3	-	-	-	-	-	-	-
Firmicutes/Mollicutes	17	-	-	-	-	-	-	-	-	-	-	-	-	-	-	-	-
Firmicutes/Bacillales	25	-	6	-	6	-	-	1	-	2	2	-	2	-	-	-	-
Firmicutes/Clostridia	38	8	20	15	3	2	-	-	-	-	-	-	-	-	-	-	-
Chlamydiae	7	-	-	-	-	-	-	-	-	-	-	-	-	-	-	-	-
Bacteroidetes	30	-	1	-	-	-	-	-	-	-	1	-	1	-	-	-	6
Chlorobi	9	7	2	-	-	-	-	-	-	-	-	-	-	-	-	-	-
Actinobacteria	40	-	2	13	-	-	-	2	1	11	-	-	1	-	-	-	17
Spirochaetes	8	-	-	1	-	-	-	-	-	-	-	-	-	-	-	-	1
Planctomycetes	3	-	-	-	-	-	-	-	-	-	-	-	-	-	-	-	-
Cyanobacteria	16	7	5	5	-	-	-	-	1	-	1	-	-	-	10	1	-
Chloroflexi	7	-	3	3	-	-	-	-	-	-	-	-	-	-	-	-	-
Deinococcus-Thermus	3	-	-	-	-	-	-	-	2	-	-	-	-	1	-	-	-
Thermotogae	6	-	1	-	-	-	-	-	-	-	-	-	-	-	-	-	-
Aquificae	2	-	-	-	-	-	-	1	-	-	-	-	-	1	-	-	-
Fusobacteria	1	-	-	-	1	-	-	-	-	-	-	-	-	-	-	-	1
Lentisphaerae	2	-	-	-	-	-	-	-	-	-	-	-	-	-	-	-	1
Verrucomicrobia	1	-	-	-	-	-	-	-	-	-	-	-	-	-	-	-	-
Candidate division TM7	3	-	-	1	-	-	-	-	-	-	-	-	-	-	-	-	-
Acidobacteria	2	1	-	-	-	-	-	-	-	-	-	-	2	-	-	-	-
Deltaproteobacteria	23	12	7	5	-	-	-	-	-	-	-	-	3	-	-	-	6
Epsilonproteobacteria	17	4	-	3	3	-	-	2	-	-	2	-	-	-	-	-	-
Alphaproteobacteria/Rickettsiales	20	-	-	-	-	-	-	-	-	-	-	-	-	-	-	-	-
Alphaproteobacteria/Others	63	7	3	5	5	-	-	3	2	2	1	-	1	11	5	11	30
Alphaproteobacteria/Rhizobiaceae	5	-	-	-	-	-	-	-	-	-	-	-	-	-	-	4	4
Betaproteobacteria/Bordetella	3	-	-	-	-	-	-	-	-	-	-	-	-	3	-	-	-
Betaproteobacteria/Burkholderiaceae	20	-	-	-	-	-	-	3	9	10	-	-	-	5	-	-	5
Betaproteobacteria/Neisseriaceae	3	-	-	-	-	-	-	-	1	-	-	-	-	1	-	-	-
Betaproteobacteria/Others	19	1	1	1	-	-	-	1	-	-	-	-	1	10	-	-	6
Gammaproteobacteria/Enterobacteriales	25	-	9	-	17	-	-	16	-	-	-	-	-	-	-	-	-
Gammaproteobacteria/Pasteurellaceae	8	3	-	-	1	-	-	-	-	-	-	-	-	-	-	-	-
Gammaproteobacteria/Vibrionaceae	12	-	-	-	1	-	-	-	-	-	-	-	-	5	-	-	-
Gammaproteobacteria/Pseudomonadaceae	8	-	-	-	1	-	-	-	-	-	-	-	-	8	-	-	6
Gammaproteobacteria/Xanthomonadaceae	5	-	-	-	-	-	-	-	-	-	-	-	-	-	-	-	-
Gammaproteobacteria/Others	62	1	3	1	4	-	-	1	-	-	1	1	1	20	-	7	4
Proteobacteria/Others	2	-	1	-	-	-	-	-	-	-	1	-	-	-	-	-	-

**Total**	**540**	**52**	**66**	**55**	**44**	**2**	**0**	**30**	**16**	**28**	**9**	**1**	**12**	**65**	**15**	**23**	**87**

*: N, number of organisms containing Ni-specific transporter; C, number of organisms containing Co-specific transporter; U, number of organisms containing transporters with unassigned function;

**: Other predicted Co transporters include CbtAB, CbtC, CbtD, CbtE, CbtF, CbtG and CbtX.

Cbi/NikMNQO transporter is the most widespread transport system for Ni and Co uptake in bacteria, which is consistent with previous observations [47]. These modular transporters belong to a novel class of ATP-dependent transporters (named energy-coupling factor or ECF transporters) that use membrane proteins to capture substrate [48]. Comparison of subunits of Cbi/NikMNQO systems in different organisms revealed that M, Q and O are universal components and are present in almost all predicted transport systems. No significant similarity was detected between NikN and CbiN, although they have similar topology (two transmembrane domains, [47]). It is known that two additional components, NikK and NikL, are involved in Ni uptake in the absence of NikN, which form the NikKMLQO system [see Additional file 4]. Phylogenetic analyses of all these components are shown [see Additional files 5, 6, 7, 8, 9, 10, 11]. In general, except for CbiO/NikO, all components showed separate Ni- and/or Co-related branches although the function of some members of these components was unclear. Almost all CbiN proteins contained the same domain (COG1930, CbiN) and had similar sequences (e-value < 0.1 based on BL2SEQ pairwise alignment). In contrast, more sequence diversity was observed for NikN, NikK and NikL proteins. Sometimes, multiple distant homologs were present in the same organism (e.g., *Desulfotalea psychrophila *and *Desulfovibrio vulgaris *contained two distantly related sequences of both NikK and NikL). Here, we divided NikN, NikK and NikL into different groups based on sequence similarity and phylogenetic analyses. Three types of NikN (named N1–N3), two of NikL (L1, L2) and three of NikK (K1–K3) were identified in bacteria. Distribution of different types of these components is shown [see Additional file 12]. Approximately 90% NikL1 co-occurred with NikK1 (the other 10% co-occurred with NikK2 or NikK3), whereas NikL2 only co-occurred with NikK2 or NikK3. Interestingly, in five proteobacteria (most are *alpha*- and *gammaproteobacteria*), such as *Rhodopseudomonas palustris *and *Shewanella sediminis*, operons for NikK1ML1QO orthologs were found to be adjacent to B_12 _biosynthesis genes or were preceded by B_12_-dependent riboswitch elements [49], implying that they are involved in Co uptake in these organisms. Phylogenies of all components showed a relatively small branch for these evolutionarily distant organisms [see Additional file 5 and Additional files 8, 9, 10, 11] although each component belonged to a large Ni-related group. These observations suggest that the Co uptake function recently evolved for NikK1ML1QO system in these organisms. However, it is not clear whether they are still involved in Ni uptake. Orphan NikK and/or NikL orthologs were also observed in several organisms which lack NikMQO but contain Ni-dependent proteins, or even lack Ni utilization (see Additional files 1, 10 and 11]. We checked their gene neighborhoods and could not find proteins directly implicating their function. Thus, they may be involved in Ni-independent pathways. In several organisms where no NikQ could be detected, a hypothetical transporter component (5 transmembrane domains, similar topology as NikQ but no sequence similarity) was always found encoded next to *nikO*. Orthologs of this hypothetical transmembrane protein were only detected in six sequenced organisms and most of them were predicted to be involved in Ni uptake [see Additional file 13], suggesting that novel Ni-related transporter component evolved in organisms lacking NikQ. In addition, different NikMs in NikMNQO or NikKMLQO system clustered in separate branches [see Additional file 5], indicating that the evolutionary process of NikM correlates with the usage of N or K+L components. However, no correlation was observed for NikM based on different subtypes of NikN, NikK and NikL components. Similarly, phylogeny of the core transporter components Q and O did not show significant similarity to that of M, N, K or L component. It should be noted that no organism that contained both NikMNQO and NikKMLQO was detected, indicating a complementary or mutually exclusive relationship between these two systems.

Two other transporter families, HupE/UreJ and NiCoT, were also found to be frequently used in bacteria (Table 2). The HupE/UreJ transporter family is widely utilized in the *Cyanobacteria *and various proteobacterial subdivisions except for the *Deltaproteobacteria *and *Epsilonproteobacteria*. Phylogenetic analysis of all collected members of this transporter family showed two separate branches of predicted Ni- and Co-specific subgroups although there were still several members with unassigned function in each branch [see Additional file 14]. The NiCoT family was detected in diverse taxonomic groups of bacteria. Compared to HupE/UreJ, NiCoT showed much more complex functional diversity and predicted Ni- and Co-specific transporters were scattered in various branches of the phylogenetic tree [see Additional file 15].

ABC transporter systems are typically major and the most active transporters of organic compounds and metals, such as zinc, manganese, amino acids and peptides. In our study, only a fraction of organisms were predicted to possess the NikABCDE system, including distant Ni ABC-type transporters identified in *Yersinia *species, YntABCDE [29]. Besides genomic context, we attempted to utilize residues which may be involved in Ni-binding (see Methods for details) to distinguish NikABCDE from homologous peptide import systems. Multiple alignment of NikA sequences and other homologs showed that most of the residues proposed to be involved in Ni-NikA interaction are conserved in predicted NikA proteins but absent in other homologs [see Additional file 16]. Except for members of the NikABCDE family in *Clostridium tetani *and *Desulfitobacterium hafniense*, which were previously predicted to be preceded by a B_12_-dependent riboswitch element [47], all NikA orthologs appeared to be Ni-specific [see Additional file 17]. Although YntA (the periplasmic Ni-binding component in the YntABCDE system) is evolutionarily distant from NikA, and it is still unclear how YntA binds Ni, gene neighborhoods could be used to identify this distant Ni ABC-type transporter family.

In addition, only 20 organisms possessed orthologs of the UreH transporter. This family was previously predicted to be Ni-specific because these genes were always located adjacent to the genes for Ni-dependent enzymes, such as urease, Ni-Fe hydrogenase and SodN [26,47]. There have been no reports that showed that UreH may also be involved in Co uptake. Here, we found that a member of the UreH family is adjacent to several B_12 _biosynthesis genes (such as CbiD and CobB), in a gammaproteobacterium, *Moritella sp. *PE36, suggesting that UreH is involved in Co uptake in this organism [see Additional file 18].

Besides the above well-characterized Ni/Co transporter families, several recently predicted Co transporters, including CbtAB, CbtC-CbtG and CbtX [31,40], were detected in 87 species, mostly in the *Proteobacteria *and *Actinobacteria *(Table 2). Essentially all of these organisms possessed the B_12 _biosynthetic pathway and many lacked known Co transporters.

In *E. coli*, the nickel repressor gene *nikR *is positioned immediately next to its target, the *nikABCDE *operon. NikR-dependent regulation was also predicted for other Ni transporters, such as NikMNQO and Ni-specific NiCoT, and Ni-dependent enzymes such as Ni-Fe hydrogenase [47]. In this study, NikR was found in less than half of the organisms containing NikABCDE, suggesting the presence of NikR-independent regulation of the NikABCDE system [see Additional files 1 and 19]). Here, the occurrence of NikR was used to supplement the searches for Ni-related transporters.

Only three Ni/Co transporter families were detected in archaea: Nik/CbiMNQO, NikABCDE, and NiCoT (Table 3). As in bacteria, Nik/CbiMNQO was the most widespread transporter system. Compared to variations in the bacterial NikMNQO and NikKMLQO systems, only NikMN1QO and NikMN2QO were detected in archaea. In contrast, the distribution of the other two transporters was not very pronounced and most NiCoT transporters did not show clear function. In the case of other predicted Co transporters, only CbtX was detected, in 7 archaeal species.

**Table 3:** Distribution of Ni/Co transporters in archaea

**Phylum**	**Total organisms**	**CbiMNQO/NikMNQO**			**NikABCDE**			**NiCoT**			**Other predicted Co transporters (CbtX)**
			
		**N**	**C**	**U**	**N**	**C**	**U**	**N**	**C**	**U**	
Nanoarchaeota	1	-	-	-	-	-	-	-	-	-	-
Crenarchaeota/Thermoproteales	6	3	-	1	-	-	-	-	-	-	-
Crenarchaeota/Desulfurococcales	4	1	-	1	-	-	-	-	-	-	-
Crenarchaeota/Sulfolobales	4	-	-	-	-	-	-	1	-	3	-
Euryarchaeota/Thermoplasmales	4	-	-	-	-	-	-	-	-	1	-
Euryarchaeota/Archaeoglobales	1	1	1	-	-	-	-	-	-	-	-
Euryarchaeota/Halobacteriales	5	2	1	2	-	-	-	-	-	-	1
Euryarchaeota/Methanosarcinales	5	5	5	1	3	-	-	-	-	-	5
Euryarchaeota/Thermococcales	4	1	-	1	-	-	-	-	-	-	-
Euryarchaeota/Methanococcales	5	1	3	5	-	-	-	-	-	-	-
Euryarchaeota/Methanopyrales	1	1	-	-	-	-	-	-	-	-	-
Euryarchaeota/Methanobacteriales	3	1	-	3	-	-	-	-	-	-	-
Euryarchaeota/Methanomicrobiales	4	4	4	-	-	-	-	-	-	1	1

**Total**	**47**	**20**	**14**	**14**	**3**	**0**	**0**	**1**	**0**	**5**	**7**

### Occurrence of Ni-dependent enzymes, B_12 _biosynthetic pathway and B12-dependent enzymes in prokaryotes

Among bacterial Ni-dependent enzymes, urease (catalyzes the hydrolysis of urea to carbon dioxide and ammonia) and Ni-Fe hydrogenase (catalyzes hydrogen evolution and uptake; it includes Ni-Fe hydrogenase I (COG0374, HyaB), Ni-Fe hydrogenase III (COG3261, HycE) and F420-reducing hydrogenase (COG3259, FrhA)) were the two most widespread families (Table 4). In the analyzed dataset, 185 organisms (58.0% of Ni-utilizing bacteria) possessed urease and 168 (52.7%) Ni-Fe hydrogenase. Occurrence of other Ni-dependent proteins was limited and mosaic (Table 4). For example, CODH/ACS, a key enzyme in the Wood-Ljungdahl pathway of anaerobic CO(2) fixation [50], was identified only in 11 organisms belonging to the *Firmicutes/Clostridia*, *Chloroflexi *and *Deltaproteobacteria*, whereas SodN was detected in 21 organisms in the *Actinobacteria*, *Bacteroidetes*, *Cyanobacteria *and some *Gammaproteobacteria*. As mentioned above (Table 1), 10 organisms containing Ni-specific transporters (mostly NikABCDE) lacked known Ni-dependent proteins. We examined the genes adjacent to the predicted transporter genes in these organisms, but did not find good candidates for Ni-dependent proteins. It is possible that these organisms possess additional Ni users which are not strictly Ni-dependent such as GlxI. We found that all these organisms containing orphan Ni transporters also contain GlxI proteins, although it is unclear which of these proteins bind Ni. Although incorrect functional assignment of some transporters (e.g., a predicted Ni-specific transporter may be involved in Co or peptide import) cannot be excluded, misassignment of function should be not significant.

**Table 4:** Distribution of Ni-dependent enzymes in bacteria

**Phylum**	**Total organisms**	**Ni-utilizing organisms**	**Organisms containing different Ni-dependent proteins**				
			
			**Urease**	**Ni-Fe hydrogenase**	**Ni-CODH**	**CODH/ACS**	**SodN**
Firmicutes/Lactobacillales	25	3	1	-	-	-	-
Firmicutes/Mollicutes	17	2	2	-	-	-	-
Firmicutes/Bacillales	25	12	9	-	-	-	-
Firmicutes/Clostridia	38	28	4	14	21	6	-
Chlamydiae	7	-	-	-	-	-	-
Bacteroidetes	30	6	2	3	-	-	2
Chlorobi	9	8	-	8	1	-	-
Actinobacteria	40	27	19	12	-	-	10
Spirochaetes	8	-	-	-	-	-	-
Planctomycetes	3	1	-	1	-	-	-
Cyanobacteria	16	13	11	10	-	-	4
Chloroflexi	7	7	1	6	-	2	-
Deinococcus-Thermus	3	1	1	-	-	-	-
Thermotogae	6	-	-	-	-	-	-
Aquificae	2	2	-	2	-	-	-
Fusobacteria	1	1	-	-	-	-	-
Lentisphaerae	2	-	-	-	-	-	-
Verrucomicrobia	1	1	1	-	-	-	-
Candidate division TM7	3	-	-	-	-	-	-
Acidobacteria	2	2	-	2	-	-	-
Deltaproteobacteria	23	20	2	17	11	3	1
Epsilonproteobacteria	17	17	4	17	2	-	-
Alphaproteobacteria/Rickettsiales	20	-	-	-	-	-	-
Alphaproteobacteria/Others	63	41	37	16	2	-	-
Alphaproteobacteria/Rhizobiaceae	5	5	5	-	-	-	-
Betaproteobacteria/Bordetella	3	3	3	-	-	-	-
Betaproteobacteria/Burkholderiaceae	20	19	19	4	-	-	-
Betaproteobacteria/Neisseriaceae	3	1	-	-	-	-	-
Betaproteobacteria/Others	19	14	12	7	-	-	-
Gammaproteobacteria/Enterobacteriales	25	23	14	18	-	-	-
Gammaproteobacteria/Pasteurellaceae	8	4	2	3	-	-	-
Gammaproteobacteria/Vibrionaceae	12	6	4	3	-	-	-
Gammaproteobacteria/Pseudomonadaceae	8	8	8	1	1	-	-
Gammaproteobacteria/Xanthomonadaceae	5	-	-	-	-	-	-
Gammaproteobacteria/Others	62	42	23	22	-	-	4
Proteobacteria/Others	2	2	1	2	-	-	-

**Total**	**540**	**319**	**185**	**168**	**38**	**11**	**21**

*: Ni-CODH, Carbon monoxide dehydrogenase; CODH/ACS, Acetyl-coenzyme A decarbonylase/synthase; SodN, superoxide dismutase SodN.

In archaea, the occurrence of these enzymes was different (Table 5). Ni-Fe hydrogenase was the most widespread protein, whereas urease was the least utilized one. SodN was not detected in archaea. In addition, the archaea-specific Ni-binding enzyme, MCR, a protein that contains a noncovalently bound Ni tetrapyrrolic cofactor (coenzyme F430) and catalyzes the final step in the biological synthesis of methane in methanogenic archaea [51], was found in all sequenced methanogens. It has been reported that MCR homologs (bind a modified F430) in some not yet cultured methanotrophic archaea (ANME) are involved in the anaerobic oxidation of methane in marine sediments [52].

**Table 5:** Distribution of Ni-dependent enzymes in archaea

**Phylum**	**Total organisms**	**Ni-utilizing organisms**	**Ni-utilizing organisms containing different Ni-dependent proteins**				
			
			**Urease**	**Ni-Fe hydrogenase**	**Ni-CODH**	**CODH/ACS**	MCR*
Nanoarchaeota	1	-	-	-	-	-	-
Crenarchaeota/Thermoproteales	6	4	-	4	-	-	-
Crenarchaeota/Desulfurococcales	4	3	-	3	-	-	-
Crenarchaeota/Sulfolobales	4	3	2	2	-	-	-
Euryarchaeota/Thermoplasmales	4	3	-	3	-	-	-
Euryarchaeota/Archaeoglobales	1	1	-	1	1	1	-
Euryarchaeota/Halobacteriales	5	3	3	-	-	-	-
Euryarchaeota/Methanosarcinales	5	5	-	3	4	5	5
Euryarchaeota/Thermococcales	4	4	-	4	-	-	-
Euryarchaeota/Methanococcales	5	5	-	5	1	5	5
Euryarchaeota/Methanopyrales	1	1	-	1	1	1	1
Euryarchaeota/Methanobacteriales	3	3	-	3	-	1	3
Euryarchaeota/Methanomicrobiales	4	4	-	4	2	2	4

**Total**	**47**	**39**	**5**	**33**	**9**	**15**	**18**

*: MCR, Methyl-coenzyme M reductase.

We also analyzed the B_12 _biosynthetic pathway in prokaryotes. By identifying key genes involved in B_12 _biosynthesis (see Methods), half of B_12_-utilizing bacteria were predicted to synthesize B_12 _and all of them contained at least one known B_12_-dependent enzyme [see Additional file 20]. The other half of B_12_-utilizing bacteria lacked the complete B_12 _biosynthetic pathway and, therefore, must be using external B_12 _via specific uptake systems, such as BtuFCD whose homologs were detected in over 90% of these organisms (see above). It was previously reported that about one-fourth of B_12_-utilizing bacteria lack the ability to synthesize B_12 _[31]. Our analysis shows that as the number of sequenced prokaryotic genomes increases, many additional organisms lacking B_12 _biosynthesis will be identified.

In order to study further the Co/B_12 _utilization in prokaryotes, we examined the occurrence of all known B_12_-dependent enzymes as means of assessing Co utilization in organisms [see Additional file 20]. Except for MGM, which was previously found in an unsequenced bacterium *Eubacterium barkeri *[53], all known B_12_-dependent proteins were detected, the most common being MetH (372 organisms), B_12_-dependent RNR II (227 organisms) and MCM (including ICM and MeaA, 212 organisms). Other proteins, including GM, 5,6-LAM, DDH, MtrA and CprA, were found only in 2 through 26 organisms.

Surprisingly, some B_12_-utilizing organisms had an extremely large number of B_12_-dependent proteins, e.g., 7 MCM members in *Nocardioides sp. JS614*, 7 CprAs and 15 different B_12_-dependent methyltransferases in *D. hafniense DCB-2*, 19 CprAs in *Dehalococcoides ethenogenes *and 32 CprAs in *Dehalococcoides sp. CBDB1 *[see Additional file 1]. Our results are consistent with previous findings which implicated these homologous enzymes in various B_12_-dependent metabolic processes [54].

We also identified 31 bacteria containing Co-binding NHases [see Additional file 20] based on the presence of Co-binding motif (CTLCSCY, [23]). All of them are B_12_-utilizing organisms and most only have single copies of NHase [see Additional file 1]. Besides, iron-containing NHases (containing CSLCSCT sequence motif, [23]) were predicted in four organisms that belong to the *Actinobacteria*, *Betaproteobacteria/Burkholderiaceae *and *Gammaproteobacteria/Others*. Phylogenetic analysis showed that these iron-containing NHases form a separate subbranch, suggesting that they might be newly evolved from Co-binding NHases [see Additional file 21].

In archaea, three-fourths of the sequenced B_12_-utilizing organisms (including all methanogens) synthesize B_12 _(Table 6). However, more than half of bacterial B12-dependent protein families were absent in archaea, including MetH, 5,6-LAM, DDH, EAL and CprA. B_12_-dependent RNR II was the most widespread B_12_-binding enzyme being present in 33 archaeal species. In addition, a variety of B_12_-dependent methyltransferases were found in archaea, most of which were present in methanogens. The *Methanosarcina *species possessed an exceptionally large number of B_12_-dependent methyltransferases, including MtaABC, MtmABC, MtbABC, MttABC, MtsABC and MtrAB (e.g., totally 15 methyltransferases in *M. acetivorans *and 12 in *M. mazei*). The presence of multiple B_12_-dependent methyltransferases involved in different pathways is clearly important for these organisms. No Co-binding NHase could be detected in archaea.

**Table 6:** Occurrence of B_12_ biosynthetic pathways and B_12_-dependent enzymes in archaea

**Phylum**	**Total organisms**	**B_12_-utilizing organisms**	**B_12 _biosynthesis pathway**	**B_12_-dependent isomerase**			**B_12_-dependent methyltransferase**	
				
				MCM/MeaA/ICM*	**GM**	**RNR II**	**Other MTs**	**MtrA**
Nanoarchaeota	1	-	-	-	-	-	-	-
Crenarchaeota/Thermoproteales	6	6	2	-	-	5	1	-
Crenarchaeota/Desulfurococcales	4	3	-	1	-	2	1	-
Crenarchaeota/Sulfolobales	4	4	4	4	-	4	-	-
Euryarchaeota/Thermoplasmales	4	4	4	4	-	4	-	-
Euryarchaeota/Archaeoglobales	1	1	1	1	-	1	1	-
Euryarchaeota/Halobacteriales	5	5	5	5	2	5	-	-
Euryarchaeota/Methanosarcinales	5	5	5	-	-	4	4	5
Euryarchaeota/Thermococcales	4	4	-	4	-	4	-	-
Euryarchaeota/Methanococcales	5	5	5	-	-	-	3	5
Euryarchaeota/Methanopyrales	1	1	1	-	-	-	-	1
Euryarchaeota/Methanobacteriales	3	3	3	-	-	1	3	3
Euryarchaeota/Methanomicrobiales	4	4	4	-	-	3	2	4

**Total**	**47**	**45**	**34**	**19**	**2**	**33**	**15**	**18**

*: MCM, methylmalonyl-CoA mutase; ICM, isobutyryl-CoA mutase; MeaA, a B_12_-dependent mutase with sequence similarity to MCM and ICM. There three subfamilies are quite similar and combined as one group in this study. GM, glutamate mutase; RNR II, B_12_-dependent ribonucleotide reductase; Other MTs, various B_12_-dependent methyltransferases such as Mta, Mtm, Mtb, Mtt, Mts, Mtv and Mtr systems.

### Prediction of a novel B_12_-dependent protein family in prokaryotes

**Figure 4:**
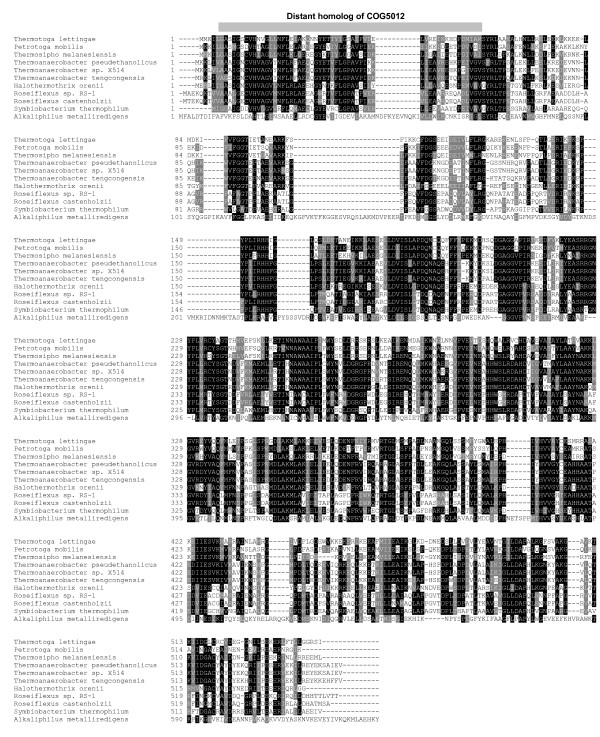
**Multiple alignment of a newly predicted B_12_-dependent protein family**. All detected sequences were used to generate the alignment. Residues shown in white on black or grey are conserved in homologs. Location of the distant homolog of B_12_-binding domain (COG5012) is indicated.

Through our analysis, a novel B_12_-dependent protein family was predicted in prokaryotes. Orthologs of this protein were detected in 11 sequenced bacteria belonging to four evolutionarily distant phyla (*Firmicutes/Clostridia*, *Firmicutes/Lactobacillales*, *Chloroflexi *and *Thermotogae*). A distant homolog of the B_12_-binding domain (COG5012, found in MetH and other methyltransferases) was detected in its N terminus (Fig. 4). Structure prediction using HHpred [55] suggested that the N-terminus may contain a TIM-barrel-like structure involved in B_12 _binding (data not shown). Analysis of genome context of this putative B_12_-dependent protein showed that it is always adjacent to NAD/NADP octopine/nopaline dehydrogenase (pfam02317), which acts on the CH-NH substrate bond using NAD(+) or NADP(+) as an acceptor. Additional enzyme candidates included D-alanine:D-alanine ligase and asparagine synthase (glutamine-hydrolyzing), which were located in the vicinity of the gene for the novel B_12_-dependent protein in several organisms. Further experiments are needed to confirm their dependence on B_12_.

### Occurrence of Ni transporters, urease and B_12_-dependent proteins in eukaryotes

Distribution of Ni transporters, urease and B_12_-dependent enzymes in eukaryotes is shown in Table 7. Except for two marine animals, *Aplysia californica *(sea slug) and *Strongylocentrotus purpuratus *(sea urchin), which contain an orphan urease, all Ni-utilizing eukaryotes contained at least one known Ni transporter and urease. However, analysis of the distribution of Ni transporters in different eukaryotic phyla showed high diversity of these proteins. NiCoT was only present in fungi (except for yeasts), whereas UreH was detected in plants (*Viridiplantae/Chlorophyta *and *Viridiplantae/Streptophyta*) and stramenopiles, and TgMTP1 was only present in land plants (*Viridiplantae/Streptophyta*). All B_12_-utilizing eukaryotes contained MetH. Except for the *Alveolata/Perkinsea *and *Viridiplantae/Chlorophyta*, all organisms also possessed MCM. RNR II was only found in *Dictyostelium discoideum *(*Dictyosteliida*) and three phytophthora species (*Stramenopiles*) and lost in fungi and animals.

**Table 7:** Distribution of Ni transporters, urease and B_12_-dependent enzymes in eukaryotes

**Phylum**	**Num. of organisms**	**Ni utilization**					**B_12 _utilization**			
		
		**Ni-utilizing organisms**	**NiCoT** **(or Nic1p)**	**UreH**	TgMTP1*	**Urease**	**B**_12_**-utilizing organisms**	**MetH**	**MCM**	**RNR II**
Cryptophyta	1	-	-	-	-	-	-	-	-	-
Diplomonadida	1	-	-	-	-	-	-	-	-	-
Parabasalidea	1	-	-	-	-	-	-	-	-	-
Kinetoplastida	5	-	-	-	-	-	3	3	3	-
Stramenopiles	4	4	-	4	-	4	4	4	4	3
Alveolata/Perkinsea	1	-	-	-	-	-	1	1	-	-
Alveolata/Apicomplexa	13	-	-	-	-	-	-	-	-	-
Alveolata/Ciliophora	2	-	-	-	-	-	-	-	-	-
Rhodophyta	1	-	-	-	-	-	-	-	-	-
Viridiplantae/Chlorophyta	3	3	-	3	-	3	3	3	-	-
Viridiplantae/Streptophyta	3	3	-	3	3	3	-	-	-	-
Dictyosteliida	1	-	-	-	-	-	1	1	1	1
Entamoebidae	3	-	-	-	-	-	-	-	-	-
Fungi/Ascomycota/Pezizomycotina	29	28	28	-	-	28	-	-	-	-
Fungi/Ascomycota/Saccharomycotina	24	-	-	-	-	-	-	-	-	-
Fungi/Ascomycota/Schizosaccharomycetes	2	2	2	-	-	2	-	-	-	-
Fungi/Basidiomycota	8	8	8	-	-	8	-	-	-	-
Fungi/Microsporidia	1	-	-	-	-	-	-	-	-	-
Fungi/Zygomycota	1	1	1	-	-	1	-	-	-	-
Metazoa/Pseudocoelomata/Nematoda	3	-	-	-	-	-	3	3	3	-
Metazoa/Coelomata/Others	2	2	-	-	-	2	2	2	2	-
Metazoa/Coelomata/Arthropoda (Insects)	19	-	-	-	-	-	-	-	-	-
Metazoa/Coelomata/Chordata/Others	10	-	-	-	-	-	10	10	10	-
Metazoa/Coelomata/Chordata/Mammals	22	-	-	-	-	-	22	22	22	-

**Total**	**160**	**51**	**39**	**10**	**3**	**51**	**49**	**49**	**45**	**4**

*: TgMTP1, Ni-related subfamily of cation-efflux family; MetH: B_12_-dependent methionine synthase.

### Evolutionary model of Ni and Co utilization

Based on the results shown above, it is possible to infer a general model of Ni and Co utilization in the three domains of life. Considering that the common property of various Ni- or Co-dependent proteins is to catalyze important reactions in the global carbon, hydrogen and nitrogen cycles, it is not surprising that both trace elements are essential for the majority of organisms. However, some organisms and even complete phyla/clades may have evolved alternative mechanisms for such reactions and are characterized by the loss of both transport systems and metalloenzymes.

Out of the five known Ni/Co transport systems in prokaryotes, only NiCoT family spans all three domains of life. If a protein family has many representatives in all domains of life and they cluster within their domains, it is thought that the family was present in the last universal common ancestor, LUCA [56,57]. We speculate that NiCoT evolved in the common ancestor of bacteria, archaea and eukaryotes. In addition, in spite of low occurrence, the presence of UreH transporter in several phyla of both bacteria and eukaryotes indicates that this family either could have been present in the last universal common ancestor but then lost in archaea, or evolved in early bacteria and was then acquired by the ancestor of eukaryotes through evolution of mitochondria. Phylogenetic analysis of UreH proteins suggested that the LUCA origin is more likely because the eukaryotic branch attaches near the bacterial root [see Additional file 18]. The B_12 _biosynthetic pathway may have evolved only in prokaryotes or has been lost in eukaryotes. In most prokaryotic phyla, organisms retained Ni and/or Co utilization traits. A complete loss of both Ni and Co utilization was only observed in two phyla, *Chlamydiae *and *Alphaproteobacteria/Rickettsiales*. We noticed that their sister phyla (such as the *Rhizobiaceae *and other *Alphaproteobacteria *for the *Rickettsiales*) commonly utilize both traits, suggesting that the loss of Ni and Co utilization happened independently in the two divisions. Considering that essentially all sequenced organisms in the two phyla were obligate intracellular parasites, it is possible that both metals are not necessary for these organisms. However, the possibility that they exploit Ni/Co-binding proteins of the host cannot be excluded.

**Figure 5:**
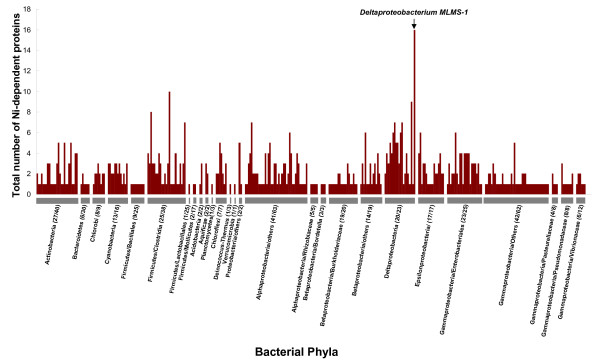
**Ni-dependent metalloproteomes in bacteria**. For each phylum, all organisms containing Ni-dependent proteins are indicated. Numbers following the name of each phylum represent the number of organisms containing a Ni-binding protein and that of total sequenced organisms, respectively. The largest Ni-dependent metalloproteome was observed in a deltaproteobacterium MLMS-1 (16 Ni-binding proteins).

**Figure 6:**
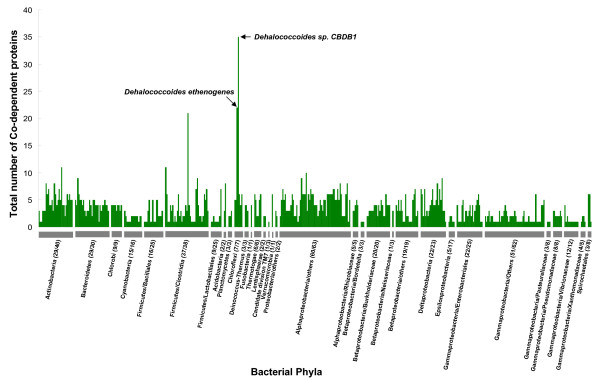
**Co-dependent metalloproteomes in bacteria**. Numbers following the name of each phylum represent the number of organisms containing at least one B_12_-binding protein and that of total sequenced organisms, respectively. The largest Co-dependent metalloproteome was observed in *Dehalococcoides sp. CBDB1 *(35 B_12_-dependent proteins, 32 of which were CprAs) and *Dehalococcoides ethenogenes *(22 B_12_-dependent proteins, 19 of which were CprAs).

**Figure 7:**
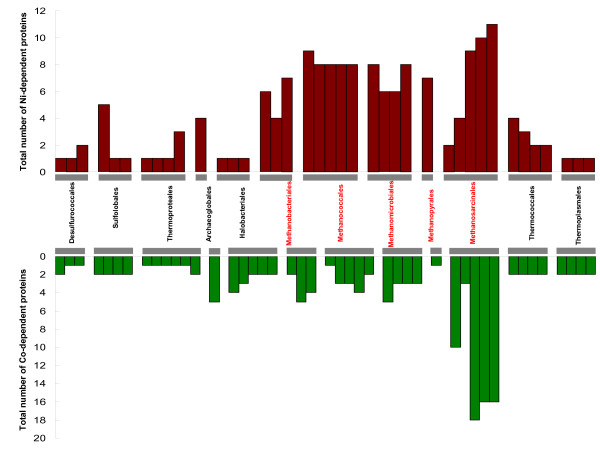
**Ni- and Co-dependent metalloproteomes in archaea**. All organisms containing Ni or Co users are shown. Methanogenic phyla are shown in red. All methanogens possess larger Ni-dependent metalloproteomes than other archaeal phyla. Only *Methanosarcina *species (*Methanosarcinales*) have large Co-dependent metalloproteomes.

Further analyses of the Ni- or Co-dependent metalloproteomes (i.e., sets of Ni- and Co(B_12_)-dependent enzymes) in different phyla provided us with an opportunity to explore the evolution of these metalloproteomes (Fig. 5, 6, 7). Normalized occurrence of these metalloproteins is shown [see Additional files 22 and 23]. There is no correlation between the number of Ni- or Co-dependent enzymes and the genome/proteome size (data not shown). In most bacteria, the size of the Ni-dependent metalloproteome was 1–4 (Fig. 5). Most of these proteins were ureases or Ni-Fe hydrogenases. However, half of sequenced *Deltaproteobacteria *appeared to have a larger Ni-dependent metalloproteome (≥ 5), including deltaproteobacterium MLMS-1, which possessed the largest Ni-dependent metalloproteome (16 Ni-binding proteins, half of which were Ni-Fe hydrogenases). Similarly, compared to most Co-utilizing species which had 1–4 Co-dependent metalloenzymes, the majority of organisms in some phyla, such as the *Chloroflexi *(including two *Dehalococcoides *species which have the largest number of B_12_-binding proteins in prokaryotes), *Spirochaetales, Actinobacteria *and *Deltaproteobacteria*, had larger Co-dependent metalloproteomes (≥ 5, Fig. 6). Therefore, the *Deltaproteobacteria *appear to be the only bacterial phylum which favors the use of both metals. In archaea, large Ni- or Co-dependent metalloproteomes were observed in methanogens (Fig. 7). Three *Methanosarcina *species in the *Methanosarcinales *phylum had the largest metalloproteomes for both Ni and Co.

A somewhat different trend was observed in eukaryotes. Few organisms utilized both Ni and Co (in the form of B_12_). Ni utilization was limited to plants and lower eukaryotes, such as fungi and stramenopiles, but was absent in vertebrates. Except for the bacterial-type NiCoT and UreH Ni transporters, additional Ni uptake systems have evolved from certain eukaryotic proteins (such as TgMTP1 in land plants). It is possible that ancient eukaryotic phyla inherited the Ni utilization trait and urease from the universal ancestor of all eukaryotes, whereas certain organisms (especially vertebrates) appeared to have lost both of them. Interestingly, urease orthologs were detected in two marine animals (*A. californica *and *S. purpuratus*) although we could not find Ni transporters in these organisms. It is unclear whether these orphan ureases still use Ni as a cofactor. Another interesting case was observed in fungi. All sequenced saccharomycotina lacked both Ni transporter and Ni-dependent urease, suggesting that this trait was lost in this fungal subgroup. Co utilization was mainly observed in animals (except for insects) and we could not detect any known B_12_-dependent proteins in most unicellular eukaryotes.

## Discussion

The importance of transition metals Ni and Co in the physiology of prokaryotes and eukaryotes is well established [1,2,10]. Both metals are essential components of several enzymes. While much effort has previously been placed on characterizing individual Ni/Co-binding proteins and the corresponding biosynthetic pathways, composition of the Co and Ni metalloproteomes and the evolutionary dynamics of utilization of these metals are largely unknown. Recently, a comparative analysis of the distribution of Ni and Co transport systems in approximately 200 microbial genomes was reported [47]. In the present study, we extended this analysis for both Ni/Co transporters and Ni/Co-dependent proteins to more than 700 bacteria, archaea and eukaryotes. Our data represent the most comprehensive analysis of genes likely to be involved in Ni and Co utilization in sequenced species.

The widespread occurrence of Ni and Co utilization traits in prokaryotes supports the idea that both metals could be used by essentially all prokaryotic phyla. Several organisms were identified that encoded Ni-dependent proteins or B_12 _biosynthetic enzymes, but did not possess known Ni or Co transporters, suggesting the presence of novel, dual-function or unspecific Ni/Co uptake systems. For example, CorA proteins are generally associated with the transport of magnesium ions but some members of the CorA family can also transport other ions such as Co and Ni [58]. Similarly, new Ni/Co-binding proteins might be present in organisms containing known transporters but not the corresponding metalloproteins.

In eukaryotes, only 9 species were identified that appeared to use both metals and most of them were unicellular organisms. Most Ni-utilizing organisms were fungi which did not utilize B_12_, whereas most B_12_-utilizing organisms were animals which lost the ability to use Ni. In addition, green algae utilized both metals, whereas land plants only possessed the Ni utilization trait. These data show that the two utilization traits have different evolutionary histories in eukaryotes, and that the acquisition or loss of each trait occurred independently in various eukaryotic phyla.

Our comparative genomic analysis showed a mosaic distribution of known Ni/Co transporters in prokaryotes. The ECF transporter Cbi/NikMNQO was the most frequently used Ni/Co uptake system in both archaea and bacteria. In contrast, the ABC transporter NikABCDE is not a common transporter in prokaryotes even though it is well characterized in *E. coli*. A recent study showed that NikA could also bind heme in *E. coli*, indicating an additional transport function independent of Ni uptake [59]. Among known Ni/Co transporters, NiCoT and UreH were the only families detected in both prokaryotes and eukaryotes. Although comparative genomic approaches allow prediction of the physiological substrate for various members of these transporters, many have unassigned function. Previous prediction of a variety of new Co transporter candidates in various microbes suggested a complex evolutionary dynamics of Co transport in prokaryotes. On the other hand, identification of different subtypes of components of NikMNQO/NikKMLQO made here also implied a complex evolutionary dynamics of Ni uptake in prokaryotes.

Analysis of Ni-dependent enzymes, B_12 _biosynthetic pathways and B_12_-dependent enzymes in prokaryotes provided a straightforward approach to analyze the distribution and evolution of Ni and Co utilization in various organisms. It should be noted that we only analyzed a set of strictly Ni- or Co-dependent proteins (for which no Ni- or Co-independent forms have been reported), which may not fully account for utilization of the two transition metals in some organisms. Indeed, a protein may potentially have different activities when binding different metals. For instance, it has been reported that in certain organisms, an aci-reductone dioxygenase has different activities when binding iron or Ni [60]. In this study, urease, the most widespread Ni-dependent enzyme in bacteria, was only detected in certain aerobic archaea. This observation was not unexpected because urease was mainly found in aerobic organisms, whereas most sequenced archaea were anaerobic. Among other Ni-dependent enzymes, superoxide dismutase SodN was essentially a bacteria-specific Ni-containing protein and MCR was specific to methanogens. In the case of Co, we detected all Co-utilizing organisms by searching for B_12_-dependent enzymes and all B_12_-producing organisms by analyzing genes involved in B_12 _biosynthesis. In bacteria, MetH was not only the most frequently used B_12_-dependent protein but also the only B_12_-binding protein in approximately 90% of organisms containing single B_12_-dependent proteins. Moreover, more than 80% of the latter organisms lacked the ability to synthesize B_12_. On the other hand, RNR II was the most abundant B_12_-dependent protein in archaea in which no MetH was observed. The observations that only half of bacterial B_12_-dependent enzymes were found in archaea and that a variety of B_12_-dependent methyltransferase families evolved in methanogens (especially in *Methanosarcina *species) implied somewhat different evolutionary trends in bacteria and archaea. It appears that B_12_-dependent methyltransferases are particularly important for metabolism of methanogenic archaea.

**Figure 8:**
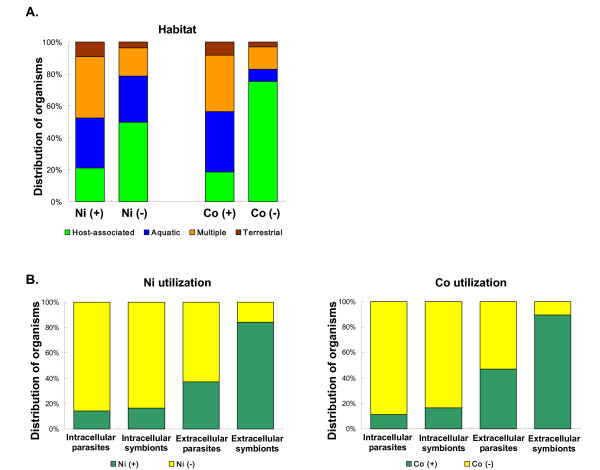
**Relationship between environmental factors and Ni/Co utilization traits in bacteria**. All organisms were classified into four groups: Ni (+), i.e., containing Ni utilization trait; Ni (-), i.e., lacking Ni utilization trait; Co (+), i.e., containing Co utilization trait; Co (-), i.e., lacking Co utilization trait. (A) Habitat; (B) Different host-associated life styles.

Previously we found that habitat, environment and other factors (e.g., oxygen requirement, optimal temperature, optimal pH and GC content) may influence the acquisition/loss of utilization traits of certain trace elements, e.g., selenium (Se) and molybdenum (Mo), in prokaryotes [61,62]. To examine the possibility that Ni and Co utilization may also be affected by some of these factors, we adopted a strategy which was previously used to analyze the evolution of Se and Mo [61,62]. First, similar to Mo utilization [62], we found that the majority of bacteria that utilized neither Ni nor Co were host-associated (i.e., parasites or symbionts, Fig. 8A), implying that host-associated life style may result in the loss of metal utilization, perhaps due to limited space and resources. Considering differences in host-associated conditions (intra- or extracellular) and the relationship between these organisms and their hosts (symbiotic or parasitic), we further divided them into four groups: obligate intracellular symbionts (6 organisms, 2 phyla), extracellular symbionts (19 organisms, 10 phyla), obligate intracellular parasites (35 organisms, 6 phyla) and extracellular parasites (113 organisms, 20 phyla). Interestingly, we found that the majority of intracellular parasites and intracellular symbionts lost the ability to utilize Ni or Co, whereas more than 80% of extracellular symbionts utilized both metals (Fig. 8B). Most obligate intracellular parasites or symbionts had much smaller genomes than extracellular organisms [see Additional file 24]. Thus, it is possible that both metal utilization traits are dispensable for intracellular organisms and hence have been lost due to the pressure on genome size, although these organisms may still depend on host Ni- or Co-dependent proteins. In contrast, the two utilization traits mostly remained intact in essentially all extracellular symbionts, presumably because they are essential to their survival.

We also observed that the genomes of Ni- and Co-utilizing organisms had a significantly higher GC content [see Additional file 25]. Organisms with low GC content (i.e., GC < 40%) which lack Ni/Co utilization were found in several phyla, most of which are intra-/extracellular parasites. Intracellular pathogens and symbionts tend to be AT rich and the higher energy cost and limited availability of G and C over A and T might be the basis for the understanding these differences [63,64]. We removed all host-associated organisms and reanalyzed the correlation with GC content, and found that the original trend disappeared (data not shown). Thus, the correlation between Ni/Co utilization and GC content indirectly reflected the loss of Ni/Co utilization in parasites.

Other factors, such as gram strain, optimal temperature and pH, also appeared to have no significant effect on evolution of either trait. In addition, no statistically significant correlation could be observed between different factors and the size of Ni- or Co-dependent metalloproteomes. In archaea, insights into dynamics of Ni and Co utilization were difficult because only a small number of archaeal genomes were sequenced and nearly all of these organisms use both metals. However, the absence of both Ni and Co utilization traits in *Nanoarchaeum equitans*, an obligate symbiont [65] with a small genome (0.49 Mbp) and low GC content (31.6%), provides further support for our observations in bacteria. In brief, host-associated life style (especially obligate intracellular) and/or small genome with low GC content may result in the loss of Ni and/or Co utilization. The requirement for both metals in prokaryotes and at the same time scattered occurrence in different phyla illustrate a dynamic nature of Ni/Co utilization.

A similar investigation of Ni and Co utilization in eukaryotes provided a first glimpse on evolutionary dynamics of Ni- and Co-dependent metabolic pathways in these organisms. The fact that most parasites used neither Ni nor Co was consistent with what we found in prokaryotes, suggesting that both metals may become unnecessary for parasites because of either reduced availability of the two trace elements or dependence on the corresponding pathways of the host. Ni utilization was mainly limited to fungi (except yeasts), land plants, green algae and stramenopiles, but it was not observed in vertebrates, nematodes, insects and yeasts which lacked both Ni transporters and urease. It is known that *S. cerevisiae *can use urea as sole nitrogen source by degrading it in two steps (catalyzed by urea carboxylase and allophanate hydrolase) to ammonia and carbon dioxide, which are independent of urease and Ni [66]. A recent study reported the identification of Ni in crystal structure of 3-hydroxyanthranilic acid 3,4-dioxygenase from *S. cerevisiae*, implying a possible presence of novel Ni-binding proteins in eukaryotes [67]. However, a crystal structure of this protein in the bacterium *Ralstonia metallidurans *showed that it binds iron instead of Ni [68], implying that this protein is not a strictly Ni-dependent protein. Considering that most prokaryotic Ni-dependent enzymes except urease are used in anaerobic metabolism and most eukaryotes require oxygen, it is possible that the use of oxygen led to the loss of Ni-dependent pathways in many eukaryotes, such that only urease was preserved and only in certain lower eukaryotes and plants. Similarly, only three bacteria-type B_12_-dependent proteins were found in eukaryotes and 90% B_12_-utilizing organisms possess only single copies of MetH and MCM. These B_12_-dependent enzymes were lost in all land plants and almost all unicellular eukaryotes including fungi, but still remain in green algae, stramenopiles and all animals with the exception of insects. However, alternative pathways, such as methionine synthesis from homocysteine by B_12_-independent MetE, have evolved in various organisms [69,70]. It should be noted that although insects and fungi appeared to have lost all known B_12_-dependent enzymes, additional Co-binding proteins have been characterized in some of these organisms. For example, certain insects (such as *Spodoptera frugiperda*) encode a Co-binding class II alpha-mannosidase [71] and *S. cerevisiae *has a Co-binding methionine aminopeptidase [72] although both proteins are activated by other metals in other organisms [73,74]. Therefore, a possibility that non-strictly specific or currently unknown Ni/Co-binding proteins or Ni/Co-containing compounds are present in organisms analyzed in this study cannot be excluded.

## Conclusion

In this study, we report a comprehensive analysis of Ni and Co utilization in prokaryotes and eukaryotes by analyzing occurrence of transporters and metal-dependent enzymes. We found that occurrence of Ni/Co transporters mostly corresponds to that of known Ni/Co-dependent proteins. A new B_12_-dependent protein family was predicted in bacteria. Most prokaryotes, including extracellular symbionts, possess the Ni/Co utilization trait, with the exception of other host-associated organisms (particularly obligate intracellular parasites and symbionts). In eukaryotes, the use of the two elements is much more restricted, with regard to the organisms that use Ni/Co, the number of Ni transporters and the number of Ni/B_12_-dependent protein families. Again, parasitic lifestyle appears to result in the loss of both utilization traits in eukaryotes.

## Methods

### Genomic sequence data

We examined fully sequenced genomes from the Entrez Genome website at NCBI. A list of fully sequenced prokaryotic and eukaryotic genomes can be found on the NCBI website [75]. Only one strain was used for each species (e.g., *E. coli *O157:H7 EDL933 was used as a representative of *E. coli*). In total, 540 bacterial, 47 archaeal, and 160 eukaryotic genomes were analyzed (as of Jun. 2008).

### Identification of Ni/Co transporters, NikR repressor, vitamin B_12 _biosynthetic pathways and Ni-/B_12_-dependent enzymes

To analyze the distribution of Ni/Co transporters, we used several well-characterized Ni/Co transport proteins (e.g., NikABCDE from *E. coli*, YntABCDE and NiCoT from *Y. pseudotuberculosis*, CbiMNQO from *S. typhimurium *and HupE from *Rhizobium leguminosarum*) and previously predicted Co transporters [31,40] as initial seed sequences to search for homologous sequences in different organisms via TBLASTN [76] with an e-value < 0.1. Additional homologs were further identified using iterative TBLASTN searches. In parallel, three cycles of PSI-BLAST with default parameters were used for the identification of distant homologs. Orthologous proteins were defined using the conserved domain (COG/Pfam/CDD) database and bidirectional best hits [77]. Considering that NikABCDE transporters have significant similarity to the ABC-type dipeptide and oligopeptide import systems [27], we also utilized the residues that were proposed to bind Ni in *E. coli *NikA as major discriminators. Residues involved in Ni binding are not well characterized and conflicting results have been reported in the literature. Cherrier et al. suggested that NikA binds Ni chelated by a small organic molecule, such as butane-1,2,4-tricarboxylate (BTC), and that some residues, including Tyr402, Arg137, Arg97 and His416, form a binding site that is involved in the BTC-Ni-NikA interaction [78]. On the other hand, Addy and coworkers showed that Ni may bind *E. coli *NikA without chelators and is bound to two histidine residues (His56 and His442, although not conserved in other NikA proteins) at a position distant from the previously characterized binding site [79]. Here, the presence of the majority of these residues was used to help predict NikA proteins. In each transporter family, subgroups specific for Ni or Co were identified based on either previous reports or gene neighborhoods (i.e., if a transporter gene in a certain organism was located adjacent to genes encoding Ni-dependent enzymes, NikR or B_12 _biosynthesis proteins, it was considered as a predicted Ni- or Co-specific transporter). Other members of detected transporter families were considered as proteins with unassigned function. It is difficult to selectively identify B_12 _transporter BtuFCD among other highly similar transport systems (such as iron/heme or siderophore transporters), although previous approaches, based on B_12 _element regulation, were utilized for the identification of BtuFCD in some bacteria [31]. Therefore, in this study, we only examined the presence of BtuFCD (or BtuBFCD in gram-negative bacteria) homologs in sequenced organisms for the possibility that the potential B_12 _uptake system is present when we could not detect B_12 _biosynthesis pathway. Orthologs of NikR were identified using a similar approach. Occurrence of B_12 _biosynthesis was verified by the presence of most of the key components involved in B_12 _biosynthetic pathway: CobE, CobF, CobG, CobM, CobN, CobS, CobT, CobW, CbiD, CbiG, CbiK and CbiX [31,80-83].

Members of known Ni-dependent protein families were also identified. In this study, Ni-dependent proteins refer to strictly Ni-binding proteins that utilize Ni as a cofactor. We excluded proteins, which may bind other metals in different organisms, such as GlxI for which the contributor to shifts in metal activation is not clear [4]. Conservation of Ni-binding ligands was also analyzed for each Ni-dependent protein and those lacking most of the ligands were discarded. Similarly, in this study, we only considered B_12_-dependent enzymes as Co-dependent proteins because of the unspecificity of metal utilization, and limited distribution and information on non-corrin Co-binding enzymes. In addition, many B_12_-dependent proteins contain multiple domains, some of which are B_12_-independent. Therefore, only B_12_-binding domain-containing proteins (most contain a conserved DXHXXG motif within the B_12_-binding region [12]) were viewed as B_12_-dependent users. A complete list of query proteins is shown [see Additional files 1, 2, 3]. The presence of Ni/Co utilization trait was then verified by the requirement for occurrence of at least one predicted Ni/Co-specific transporter, or B_12 _biosynthesis trait, or at least one Ni/Co-dependent enzyme. Protein sequences for transporters and users collected in this study are provided [see Additional files 26 and 27].

### Multiple sequence alignment and phylogenetic analysis

A recently reconstructed phylogenetic tree was adopted to analyze the distribution of organisms that utilize Ni/Co in different taxonomies [84]. This tree of life was based on concatenation of 31 orthologs (most are ribosomal proteins) occurring in 191 species with sequenced genomes. The use of a common protein set across all three domains of life enables an objective, quantitative analysis of the consistency of traditional taxonomic groupings. Multiple sequence alignments were performed using CLUSTALW [85] with default parameters and ambiguous alignments in highly variable regions were excluded. Phylogenetic trees were reconstructed by PHYLIP programs [86]. Pairwise distance matrices were calculated by PROTDIST to estimate the expected amino acid replacements per position. Neighbor-joining trees were obtained with NEIGHBOR and the most parsimonious trees were determined with PROTPARS. To evaluate robustness of the trees, we performed maximum likelihood (ML) with PHYML [87] using default parameters and likelihood test. If inconsistent topologies were obtained, a third program MrBayes [88], a Bayesian estimation of phylogeny, was used. The final phylogenetic tree was then manually refined for visualization purposes.

## Abbreviations

Ni: nickel; Co: cobalt; Ni-CODH: Ni-containing carbon monoxide dehydrogenase; CODH/ACS: acetyl-coenzyme A decarbonylase/synthase; SodN: Ni-containing superoxide dismutase; MCR: methyl-coenzyme M reductase; GlxI: glyoxalase I; MCM: methylmalonyl-CoA mutase; ICM: isobutyryl-CoA mutase; GM: glutamate mutase; MGM: methyleneglutarate mutase; 5,6-LAM: D-lysine 5,6-aminomutase; RNR II: B_12_-dependent ribonucleotide reductase; DDH: diol dehydratase; EAL: ethanolamine ammonia lyase; MetH: B_12_-dependent methionine synthase; CprA: reductive dehalogenases; NHase: nitrile hydratase; ABC: ATP-binding cassette; ECF: energy-coupling factor; TgMTP1: Ni-related subfamily of cation-efflux family; HyaB: Ni-Fe hydrogenase I; HycE: Ni-Fe hydrogenase III; FrhA: F420-reducing hydrogenase; Se: selenium; Mo: molybdenum; ML: maximum likelihood; LUCA: last universal common ancestor.

## Authors' contributions

YZ and VNG designed the study. YZ carried out computational studies, including comparative genomics, sequence alignment, phylogenetic analysis and drafted the manuscript. DAR, MSG and VNG analyzed the data and edited the manuscript. All authors read and approved the final manuscript.

## Supplementary Material

Additional file 1 **Ni/Co utilization in bacteria.** The table shows the distribution of Ni/Co transporters, Ni-dependent proteins, B_12_-biosynthesis pathway proteins and Co/B_12_-dependent proteins in bacteria. Click here for file

Additional file 2 **Ni/Co utilization in archaea.** The table shows the distribution of Ni/Co transporters, Ni-dependent proteins, B_12_-biosynthesis pathway proteins and B_12_-dependent proteins in archaea. Click here for file

Additional file 3 **Ni/Co utilization in eukaryotes.** The table shows the distribution of Ni transporters, Ni-dependent proteins and B_12_-dependent proteins in sequenced eukaryotes. Click here for file

Additional file 4 **Topology of protein components of CbiMNQO, NikMNQO and NikKMLQO systems.** This figure shows common components of Ni and Co uptake, including CbiM/NikM, CbiQ/NikQ and CbiO/NikO. CbiN is specific for Co uptake, and NikN and NikK/NikL for Ni uptake. Based on sequence similarity, NikN, NikK and NikL were divided into different subtypes. Click here for file

Additional file 5 **Phylogenetic analysis of CbiM/NikM.** Predicted NikM and CbiM proteins are shown in red and green, respectively. Other members with unclear function are shown in black. Two separate branches for NikM in either NikMNQO or NikKMLQO system are also shown. NikM homologs in five proteobacteria which are predicted to be involved in Co uptake based on gene neighborhood are also shown in green. Click here for file

Additional file 6 **Phylogenetic analysis of CbiN.** Predicted CbiN proteins are shown in green and others in black. Click here for file

Additional file 7 **Phylogenetic analysis of NikN.** Predicted NikN proteins are shown in red and others in black. Separate branches for different subtypes of NikN are also shown. Click here for file

Additional file 8 **Phylogenetic analysis of CbiQ/NikQ.** Predicted NikQ and CbiQ proteins are shown in red and green, respectively. Other members with unclear function are shown in black. Click here for file

Additional file 9 **Phylogenetic analysis of CbiO/NikO.** Predicted NikO and CbiO proteins are shown in red and green, respectively. Other members with unclear function are shown in black. Click here for file

Additional file 10 **Phylogenetic analysis of NikK.** Predicted NikK proteins are shown in red and those with unclear function in black. Separate branches for three subtypes of NikK are also shown. NikK1 homologs in five proteobacteria which are predicted to be involved in Co uptake based on gene neighborhood are shown in green. Organisms containing NikK homologs but lacking NikMQO are shown in black and italic. Click here for file

Additional file 11 **Phylogenetic analysis of NikL. **Predicted NikL proteins are shown in red and those with unclear function in black. Separate branches for three subtypes of NikL are also shown. NikL1 homologs in five proteobacteria which are predicted to be involved in Co uptake based on gene neighborhood are shown in green. Organisms containing NikL homologs but lacking NikMQO are shown in black and italic. Click here for file

Additional file 12 **Distribution of different types of NikN, NikL and NikK in bacteria.** Three types of NikN, two of NikL and three of NikK were identified based on sequence similarity. Click here for file

Additional file 13 **Multiple alignment of a permease-like protein.** This protein was only detected in six sequenced Ni-utilizing organisms. Its gene is always located within the NikMNO operon which is involved in Ni uptake. Click here for file

Additional file 14 **Phylogenetic analysis of HupE/UreJ. **Predicted Ni- and Co-specific transporters are shown in red and green, respectively. Other members with unclear function are shown in black. Click here for file

Additional file 15 **Phylogenetic analysis of NiCoT.** Predicted Ni- and Co-specific transporters are shown in red and green, respectively. Other members with unclear function are shown in black. As inconsistent topologies were derived from PHYLIP and PHYML, MrBayes was used for tree construction and 100,000 trees were generated with a sample frequency of 100 and a total of 1000 trees. The bootstrap values are shown on the branch forks. Click here for file

Additional file 16 **Multiple alignment of NikA and other homologs. **Residue sets proposed to be involved in Ni-binding in *E. coli *by various groups are shown in different colors. Ligands suggested by Cherrier et al. are highlighted in red background and those suggested by Addy et al. in blue background. Other residues shown in white on black or grey are conserved in homologs. Click here for file

Additional file 17 **Phylogenetic analysis of NikA and other homologs.** Predicted Ni- and Co-specific NikAs are shown in red and green, respectively. Other Ni-unrelated homologs are shown in black. Click here for file

Additional file 18 **Phylogenetic analysis of UreH.** Predicted Ni- and Co-specific transporters are shown in red and green, respectively. Other members with unclear function are shown in black. Click here for file

Additional file 19 **Phylogenetic analysis of NikR.** Ni-utilizing organisms are shown in red. Organisms in which *nikR *gene is located very close to that of either a Ni-related transporter or a Ni-dependent enzyme are shaded. Organisms which do not utilize Ni are shown in black. Click here for file

Additional file 20 **Occurrence of the B_12 _biosynthetic pathway and Co/B_12_-dependent enzymes in bacteria.** This table shows a summary of the occurrence of the B_12 _biosynthetic pathway and Co/B_12_-dependent enzymes in various bacterial phyla. Click here for file

Additional file 21 **Phylogenetic analysis of NHases.** Predicted Co- and iron-containing NHase proteins are shown in green and brown, respectively. Click here for file

Additional file 22 **Normalized occurrence of Ni- and Co-dependent proteins in bacteria.** Each column shows a fraction of Ni- or Co-dependent proteins detected relative to the total number of annotated proteins for each organism. Numbers following the name of each phylum represent the number of organisms containing at least one user and that of total sequenced organisms, respectively. (A) Ni; (B) Co. Organisms with the largest Ni- or Co-dependent metalloproteomes are also shown. Click here for file

Additional file 23 **Normalized occurrence of Ni- and Co-dependent proteins in archaea.** Each column shows a fraction of Ni- or Co-dependent proteins detected relative to the total number of annotated proteins for each organism. Numbers following the name of each phylum represent the number of organisms containing at least one user and that of total sequenced organisms, respectively. Click here for file

Additional file 24 **Distribution of genome size in different host-associated organisms.** All host-associated organisms were divided into four groups: intracellular symbionts (6 organisms), extracellular symbionts (19 organisms), intracellular parasites (35 organisms) and extracellular parasites (113 organisms). Each column shows the genome size of each organism in various groups. Numbers following the name of group represent the average value of genome size in each group. Click here for file

Additional file 25 **Relationship between GC content and Ni/Co utilization traits in bacteria.** All organisms were classified into four groups: Ni (+), i.e., containing Ni utilization trait; Ni (-), i.e., lacking Ni utilization trait; Co (+), i.e., containing Co utilization trait; Co (-), i.e., lacking Co utilization trait. Click here for file

Additional file 26 **Ni/Co-associated sequences in bacteria.** The file contains sequences of Ni/Co transporters and metalloproteins in prokaryotes. Click here for file

Additional file 27 **Ni/Co-associated sequences in eukaryotes.** The file contains sequences of Ni/Co transporters and metalloproteins in representative eukaryotes. Click here for file
