# Supplementary figures and images for: Comparative genomic analyses of nickel, cobalt and vitamin B12 utilization
Source: BMC Genomics. 2009 Feb 10;10:78. doi: 10.1186/1471-2164-10-78 (PMC2667541; doi:10.1186/1471-2164-10-78)

Common  
components  
for Ni and Co  
uptake

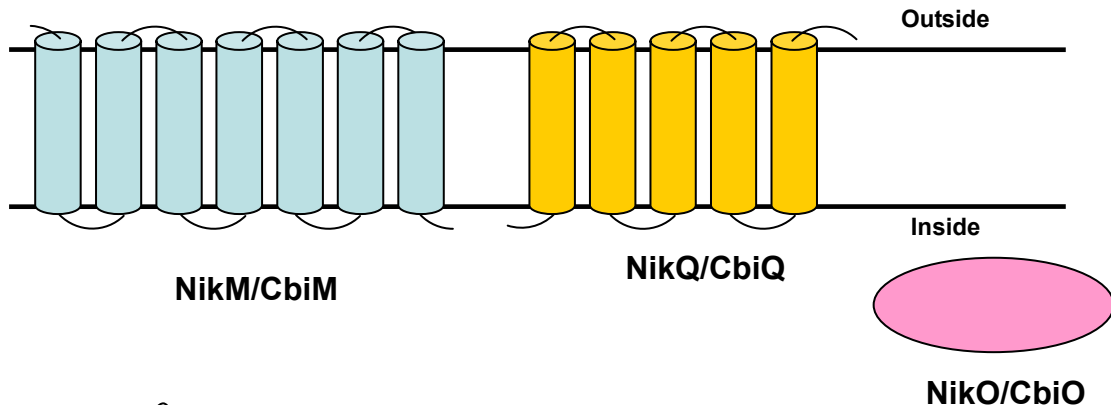

Co uptake  
(CbiMNQO)

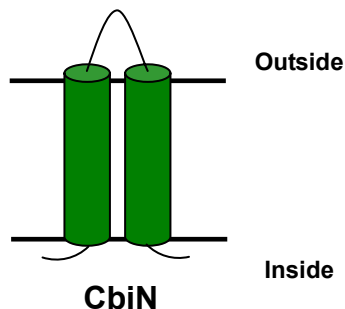

Ni uptake  
(NikMNQO or  
NikKMLQO)

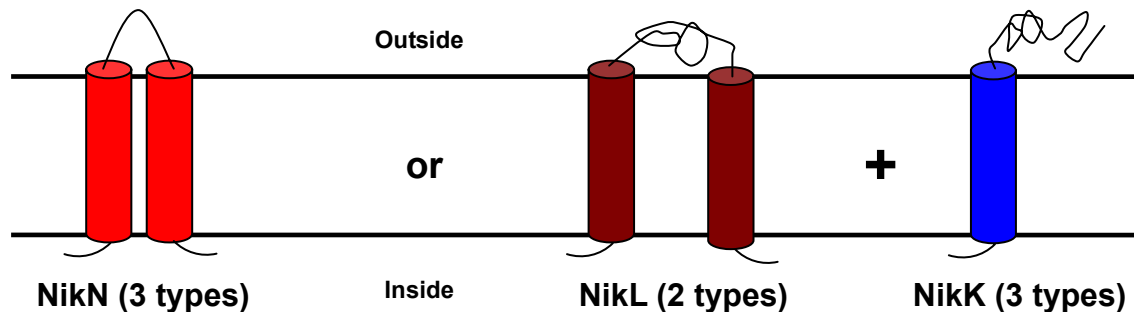

Supplement: Additional file 4 — Topology of protein components of CbiMNQO, NikMNQO and NikKMLQO systems. This figure shows common components of Ni and Co uptake, including CbiM/NikM, CbiQ/NikQ and CbiO/NikO. CbiN is specific for Co uptake, and NikN and NikK/NikL for Ni uptake. Based on sequence similarity, NikN, NikK and NikL were divided into different subtypes. [file 1471-2164-10-78-S4.pdf]

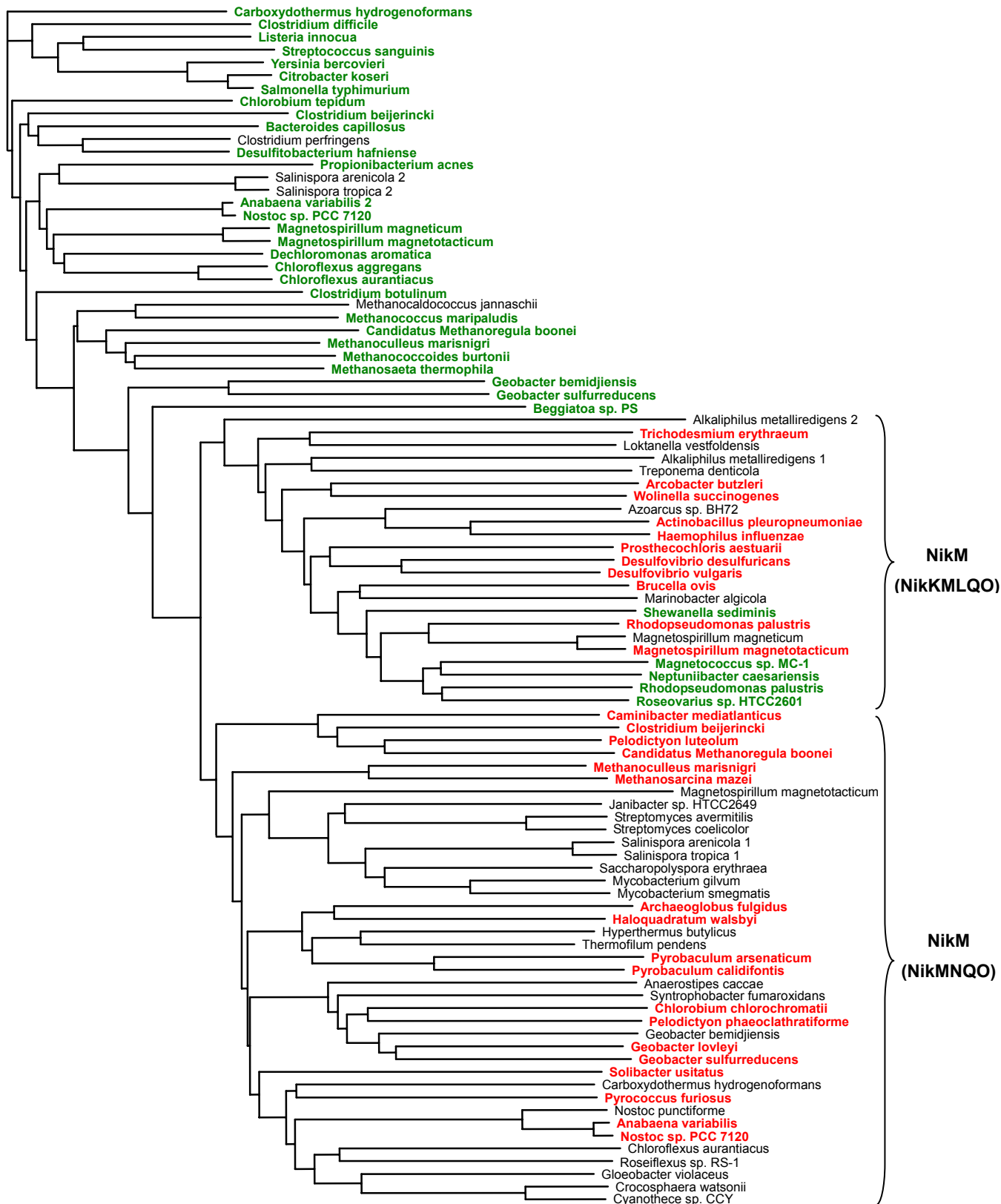

Supplement: Additional file 5 — Phylogenetic analysis of CbiM/NikM. Predicted NikM and CbiM proteins are shown in red and green, respectively. Other members with unclear function are shown in black. Two separate branches for NikM in either NikMNQO or NikKMLQO system are also shown. NikM homologs in five proteobacteria which are predicted to be involved in Co uptake based on gene neighborhood are also shown in green. [file 1471-2164-10-78-S5.pdf]

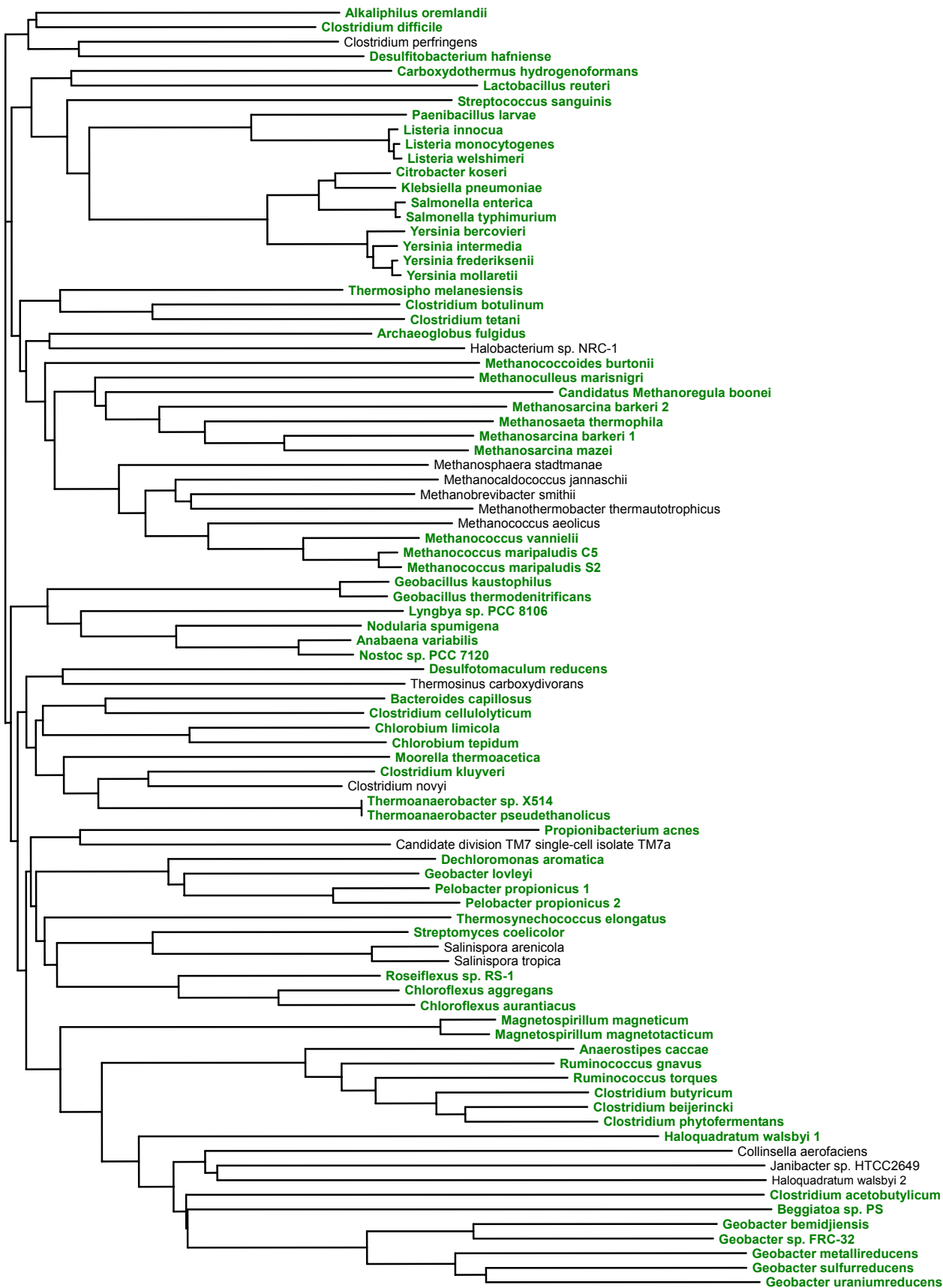

Supplement: Additional file 6 — Phylogenetic analysis of CbiN. Predicted CbiN proteins are shown in green and others in black. [file 1471-2164-10-78-S6.pdf]

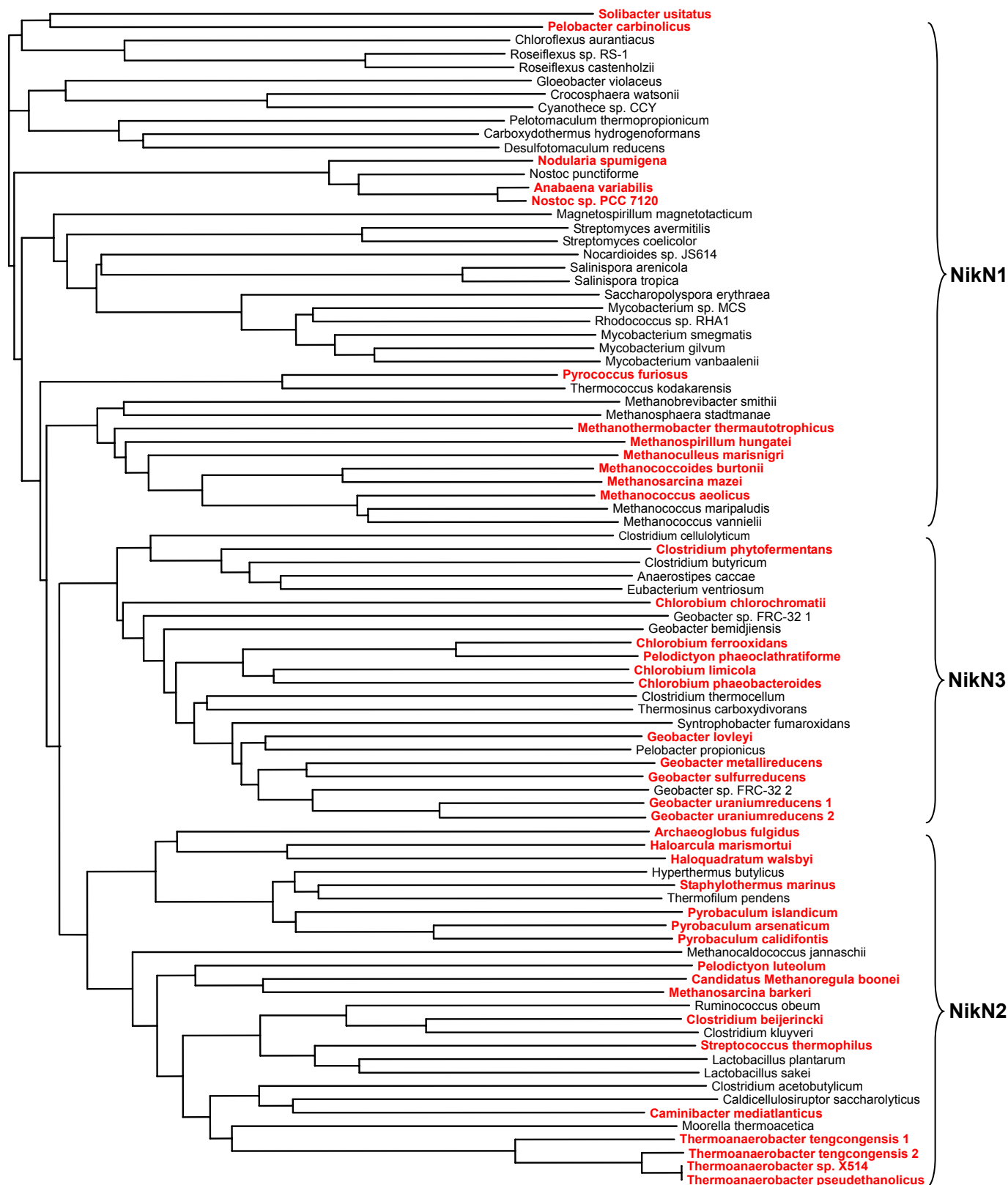

Supplement: Additional file 7 — Phylogenetic analysis of NikN. Predicted NikN proteins are shown in red and others in black. Separate branches for different subtypes of NikN are also shown. [file 1471-2164-10-78-S7.pdf]

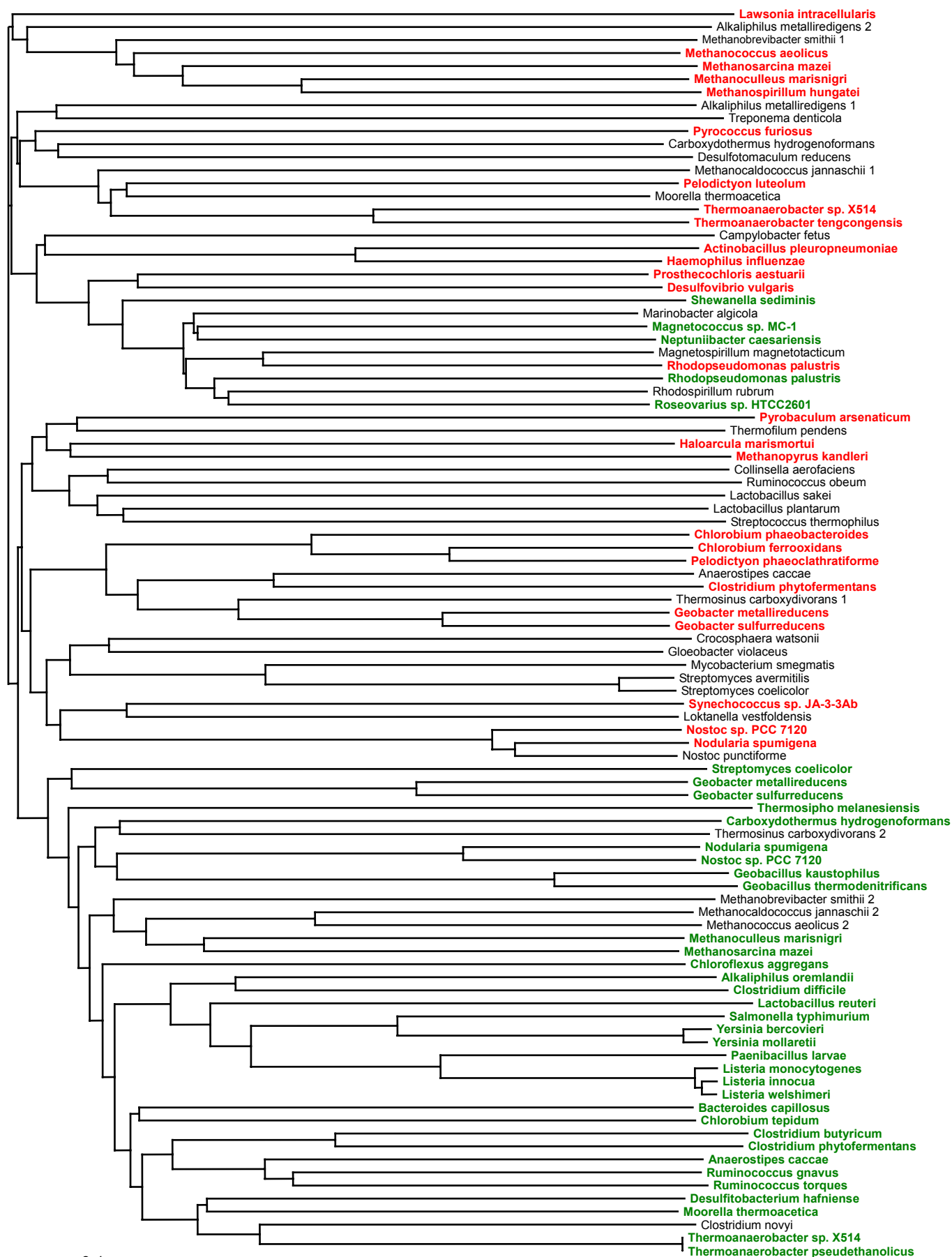

0.1

Supplement: Additional file 8 — Phylogenetic analysis of CbiQ/NikQ. Predicted NikQ and CbiQ proteins are shown in red and green, respectively. Other members with unclear function are shown in black. [file 1471-2164-10-78-S8.pdf]

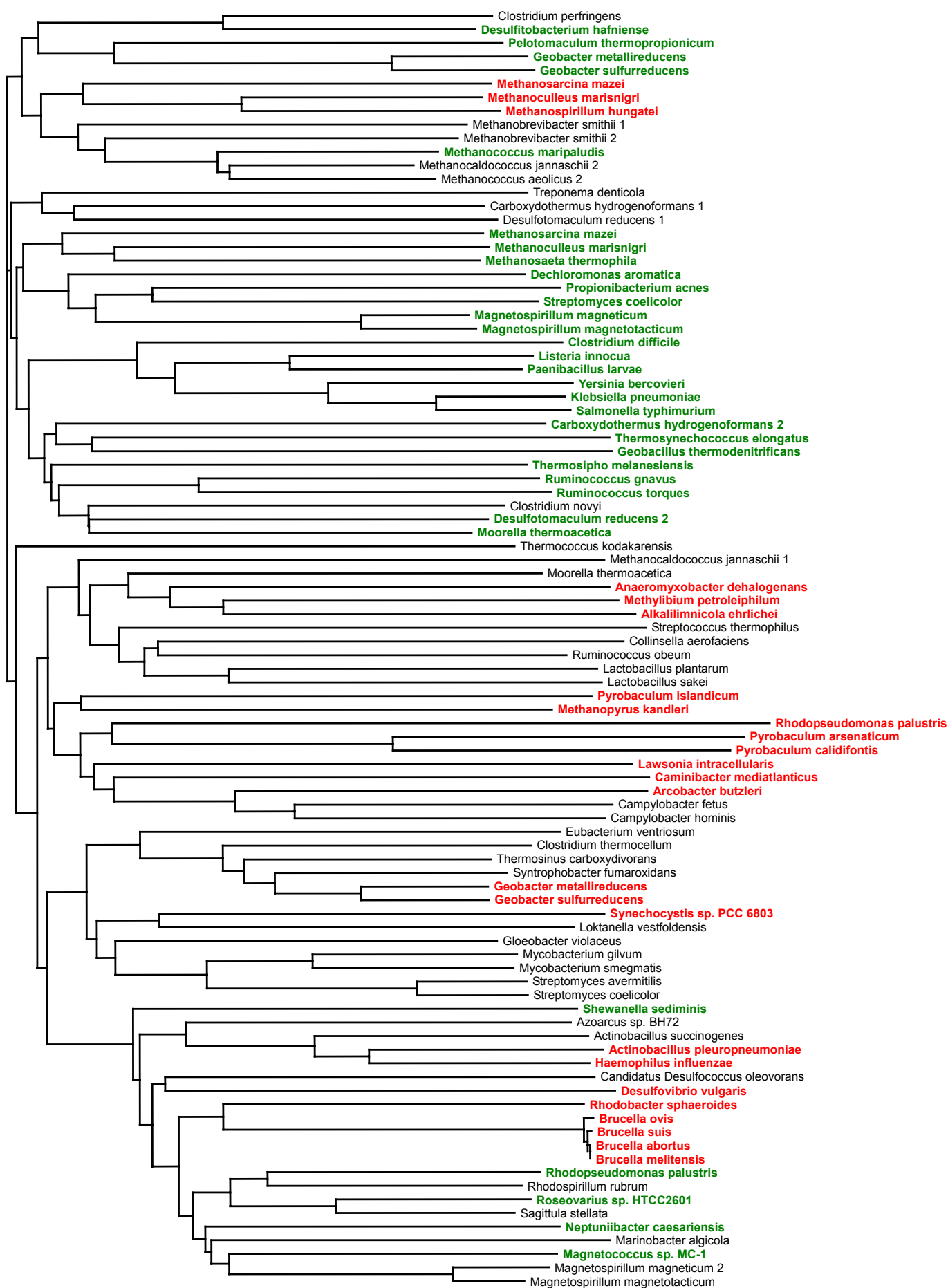

Supplement: Additional file 9 — Phylogenetic analysis of CbiO/NikO. Predicted NikO and CbiO proteins are shown in red and green, respectively. Other members with unclear function are shown in black. [file 1471-2164-10-78-S9.pdf]

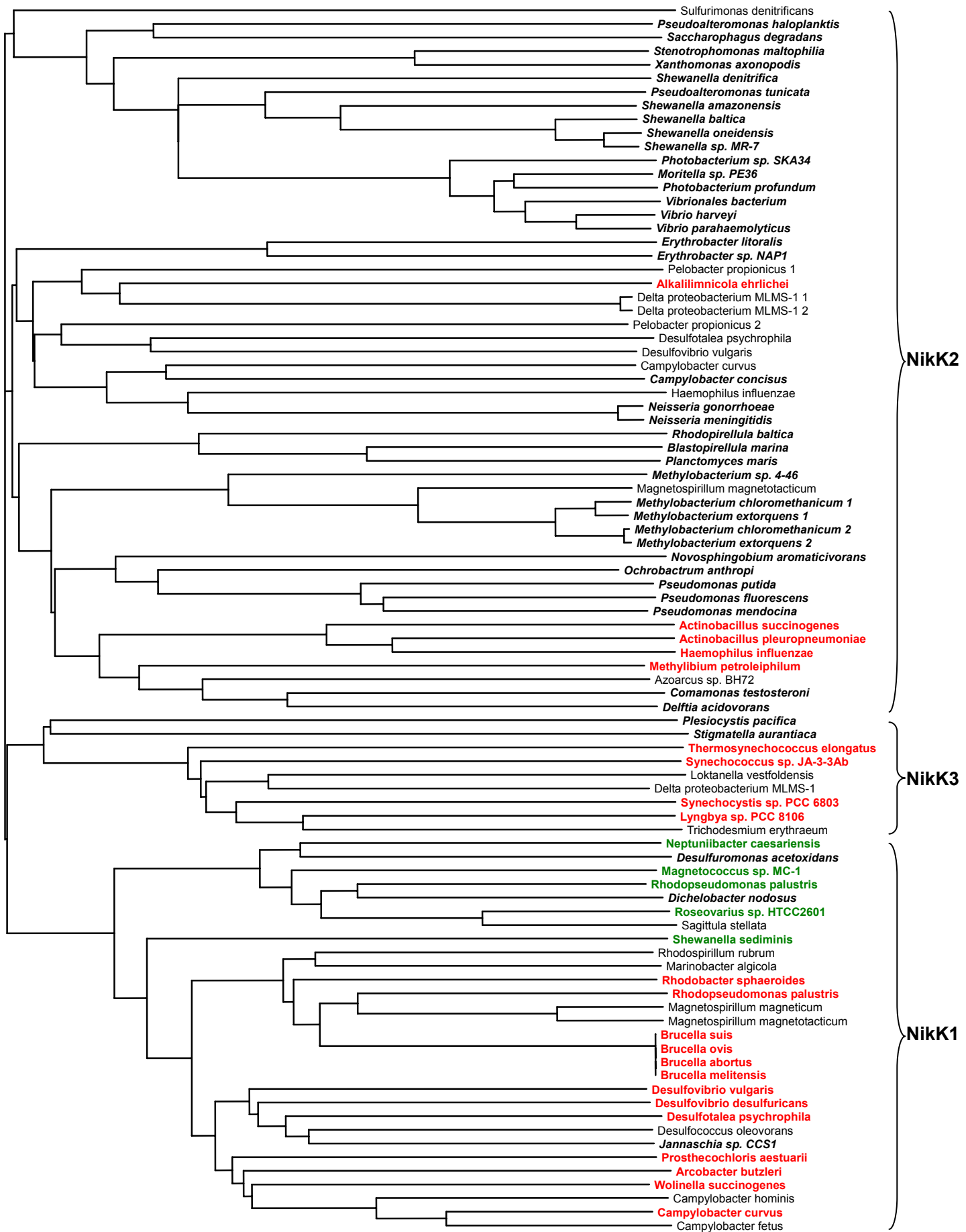

Supplement: Additional file 10 — Phylogenetic analysis of NikK. Predicted NikK proteins are shown in red and those with unclear function in black. Separate branches for three subtypes of NikK are also shown. NikK1 homologs in five proteobacteria which are predicted to be involved in Co uptake based on gene neighborhood are shown in green. Organisms containing NikK homologs but lacking NikMQO are shown in black and italic. [file 1471-2164-10-78-S10.pdf]

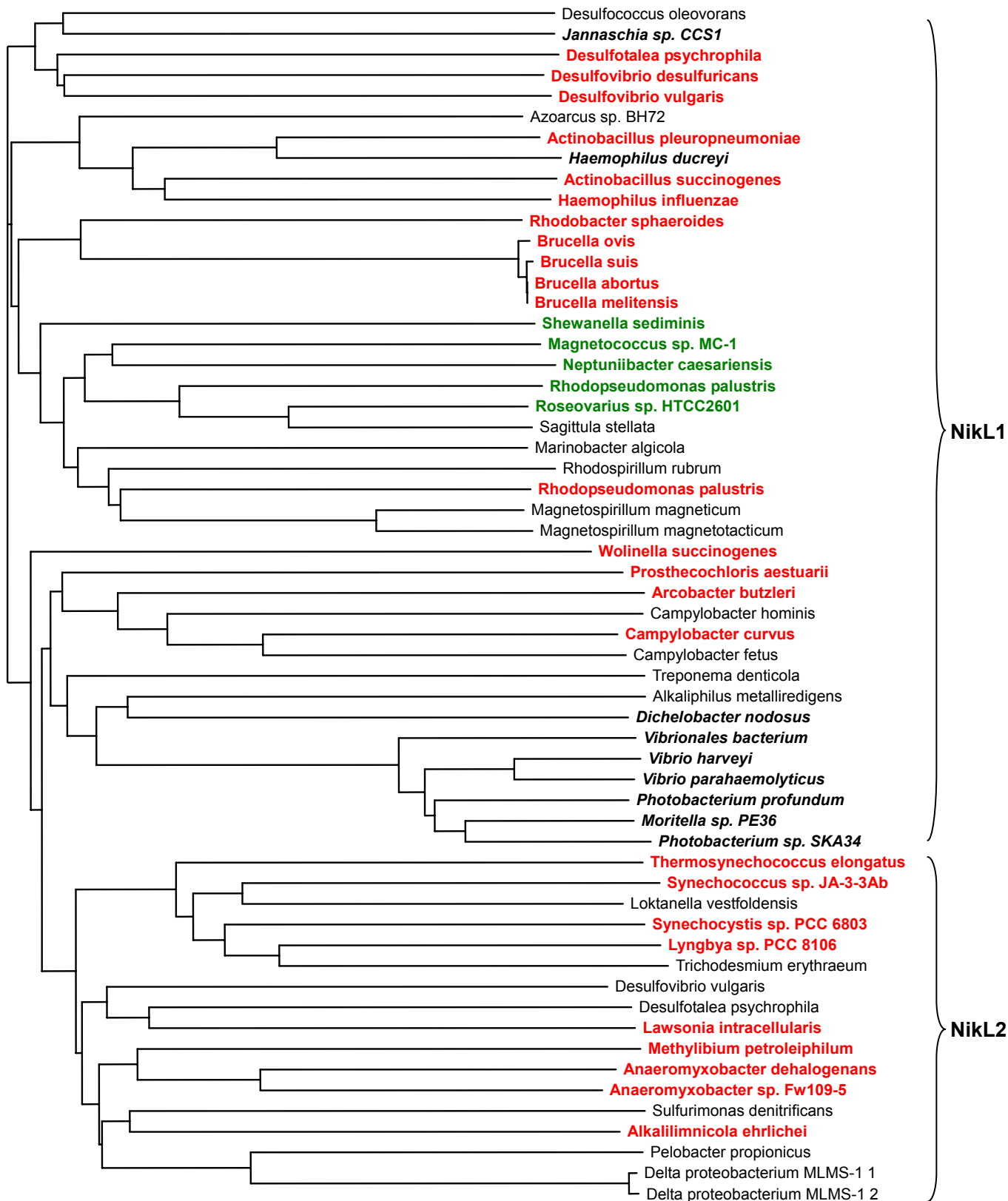

Supplement: Additional file 11 — Phylogenetic analysis of NikL. Predicted NikL proteins are shown in red and those with unclear function in black. Separate branches for three subtypes of NikL are also shown. NikL1 homologs in five proteobacteria which are predicted to be involved in Co uptake based on gene neighborhood are shown in green. Organisms containing NikL homologs but lacking NikMQO are shown in black and italic. [file 1471-2164-10-78-S11.pdf]

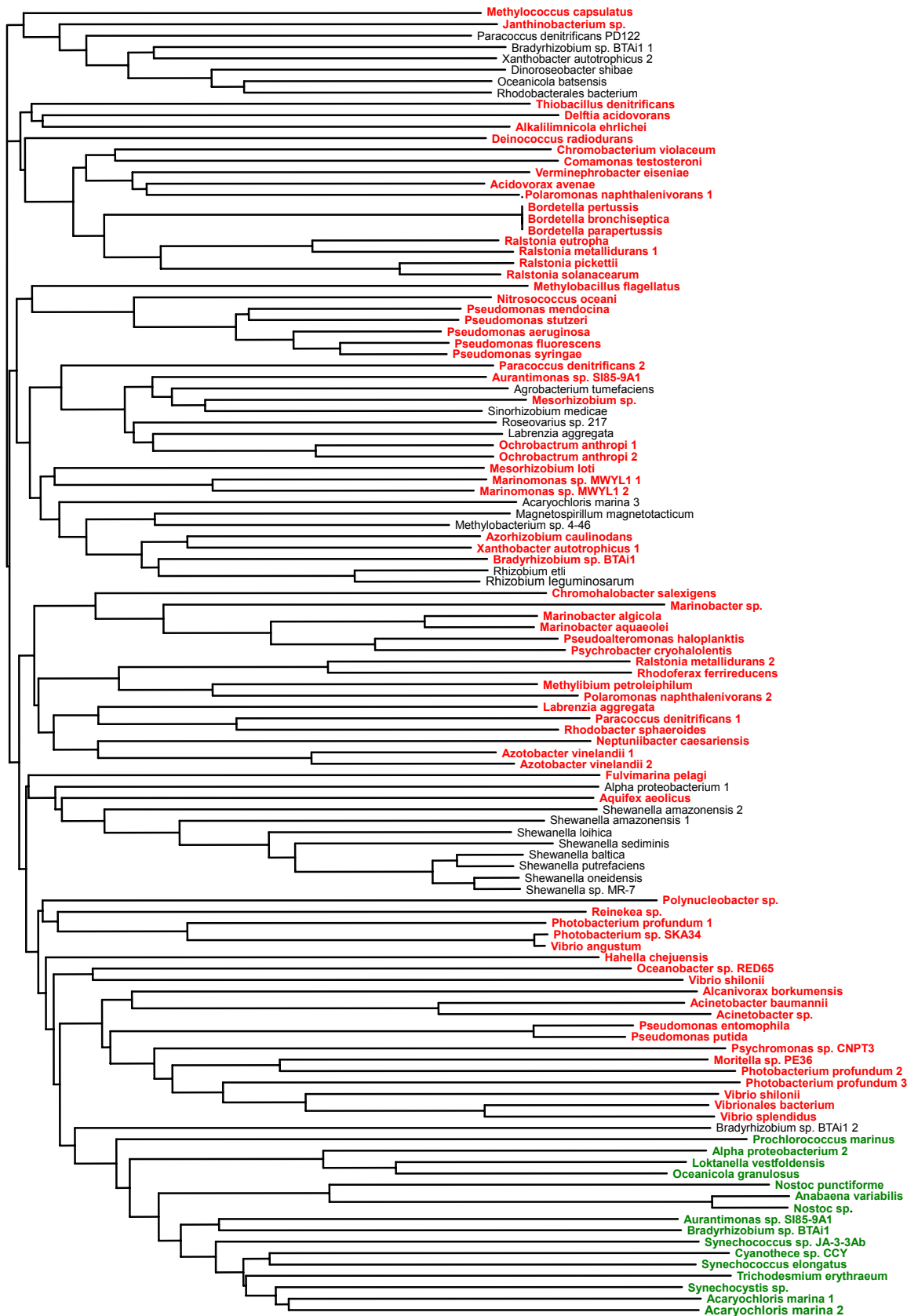

Supplement: Additional file 14 — Phylogenetic analysis of HupE/UreJ. Predicted Ni- and Co-specific transporters are shown in red and green, respectively. Other members with unclear function are shown in black. [file 1471-2164-10-78-S14.pdf]

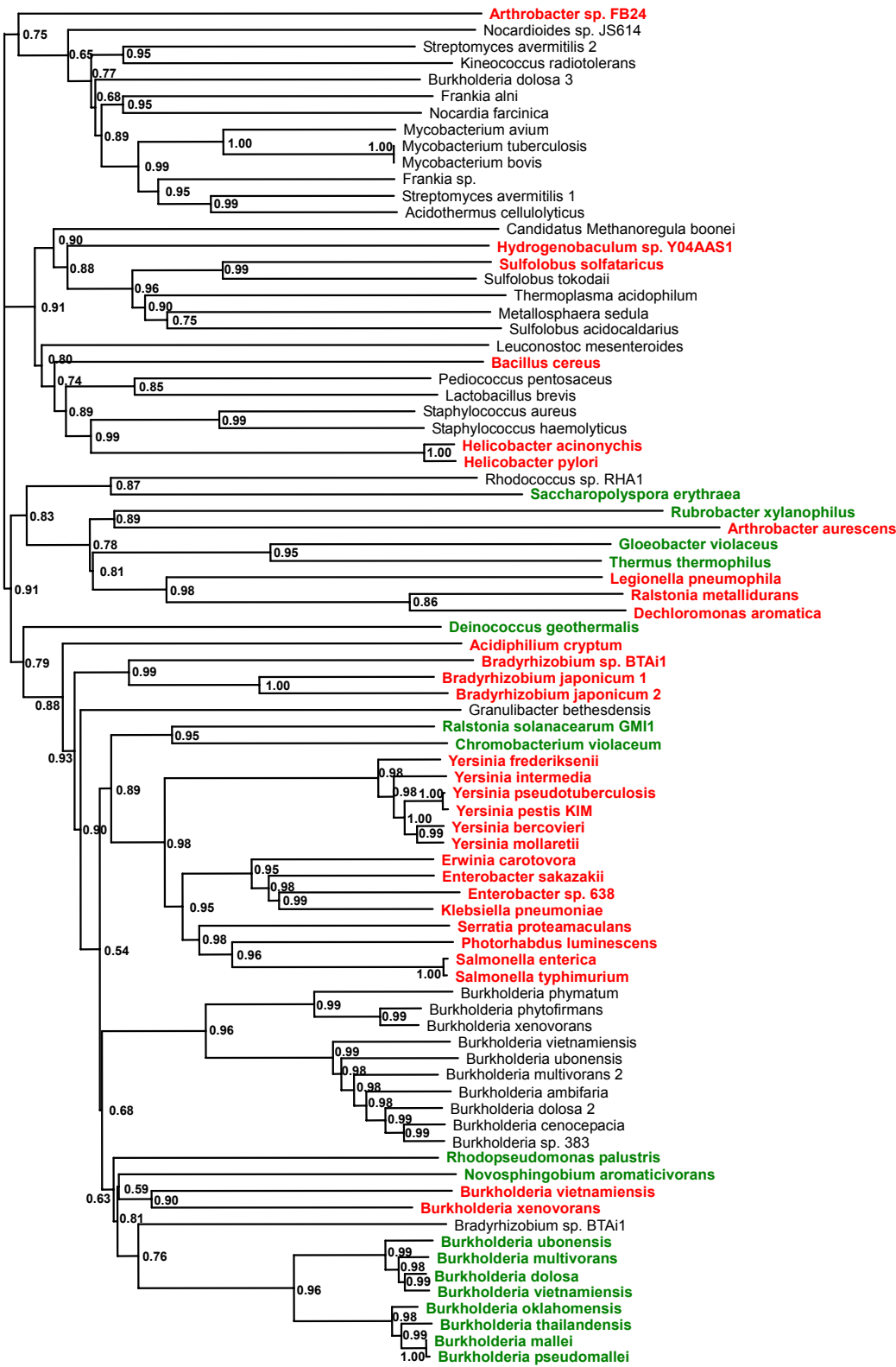

Supplement: Additional file 15 — Phylogenetic analysis of NiCoT. Predicted Ni- and Co-specific transporters are shown in red and green, respectively. Other members with unclear function are shown in black. As inconsistent topologies were derived from PHYLIP and PHYML, MrBayes was used for tree construction and 100,000 trees were generated with a sample frequency of 100 and a total of 1000 trees. The bootstrap values are shown on the branch forks. [file 1471-2164-10-78-S15.pdf]

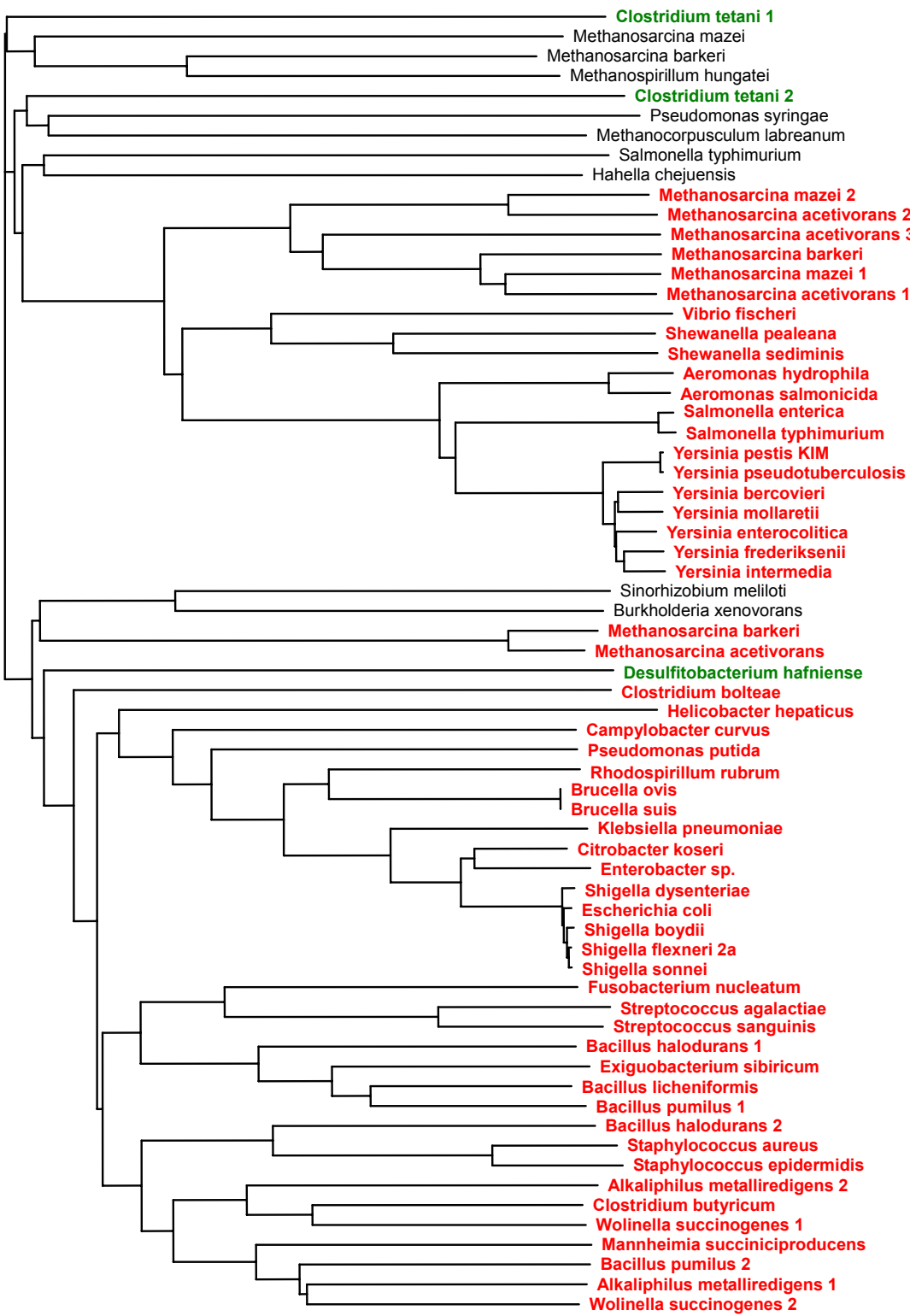

0.1

Supplement: Additional file 17 — Phylogenetic analysis of NikA and other homologs. Predicted Ni- and Co-specific NikAs are shown in red and green, respectively. Other Ni-unrelated homologs are shown in black. [file 1471-2164-10-78-S17.pdf]

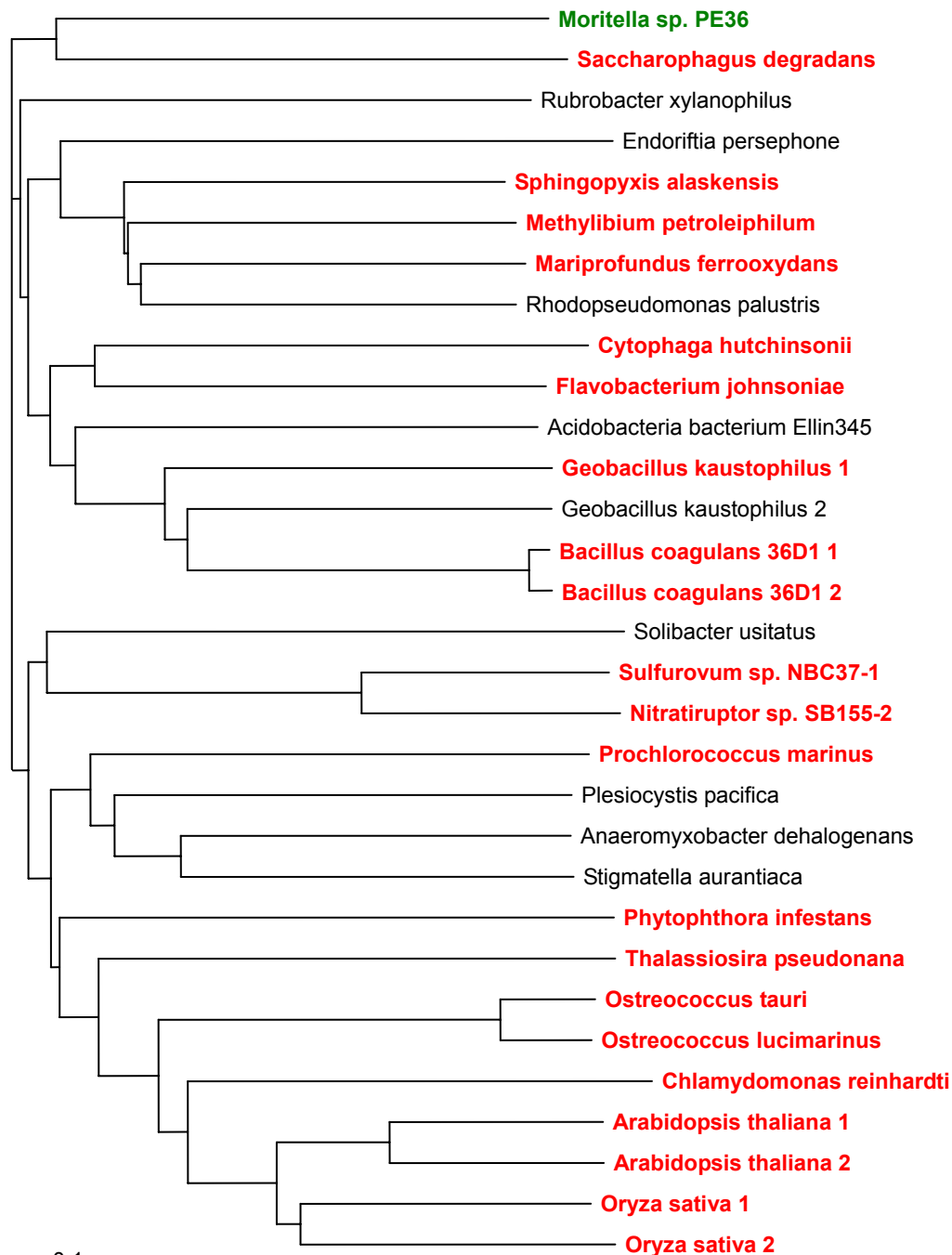

Supplement: Additional file 18 — Phylogenetic analysis of UreH. Predicted Ni- and Co-specific transporters are shown in red and green, respectively. Other members with unclear function are shown in black. [file 1471-2164-10-78-S18.pdf]

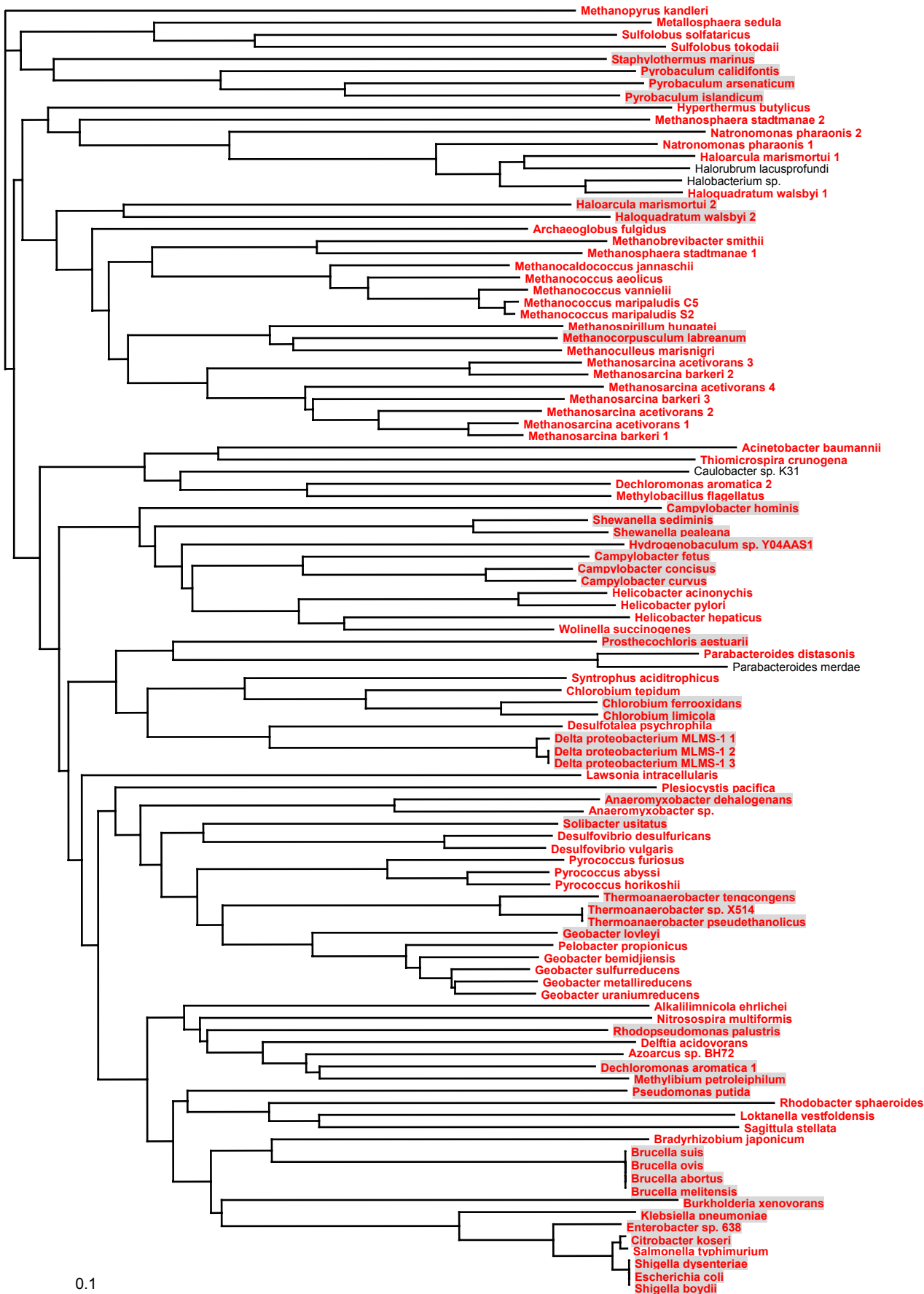

Supplement: Additional file 19 — Phylogenetic analysis of NikR. Ni-utilizing organisms are shown in red. Organisms in which nikR gene is located very close to that of either a Ni-related transporter or a Ni-dependent enzyme are shaded. Organisms which do not utilize Ni are shown in black. [file 1471-2164-10-78-S19.pdf]

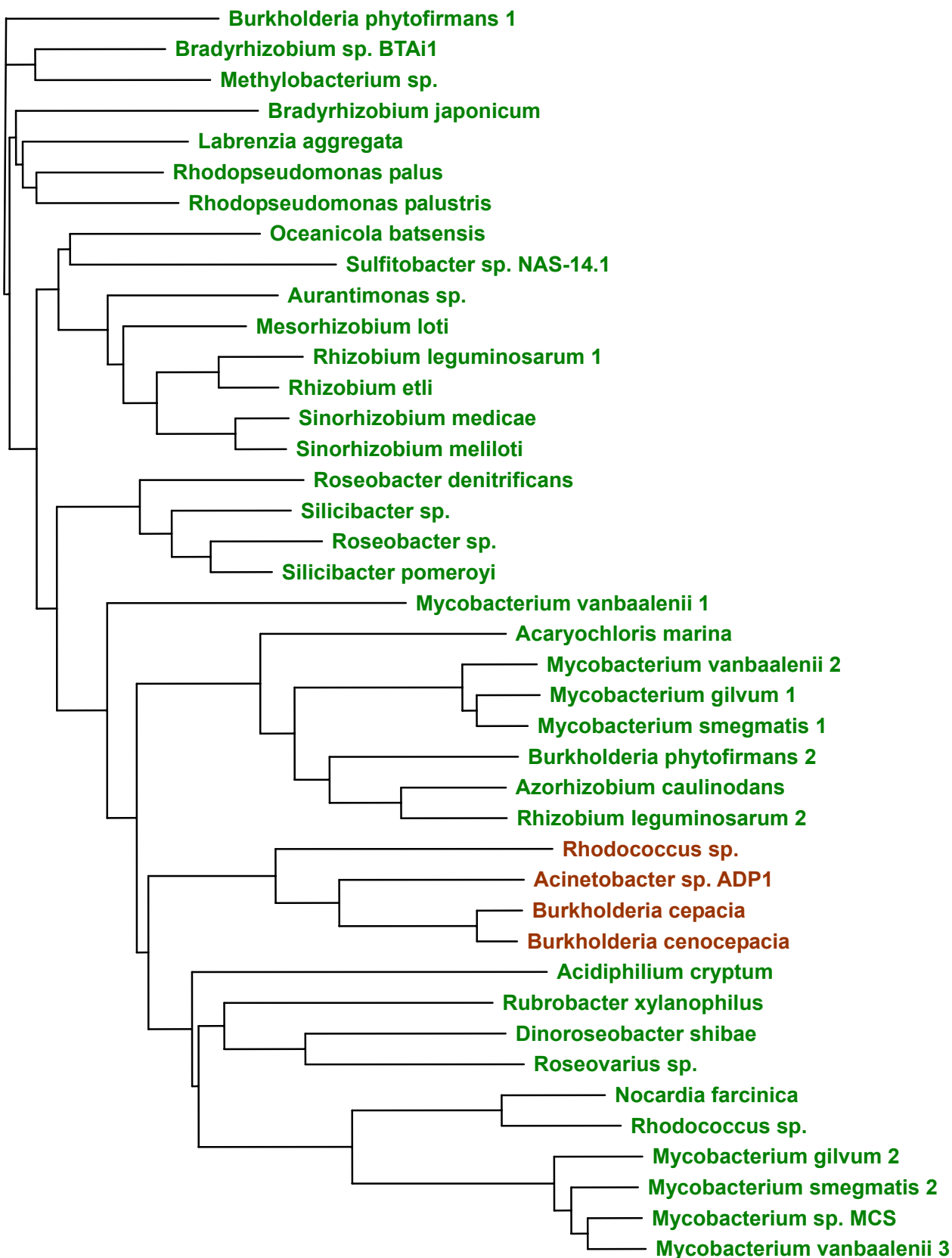

0.1

Supplement: Additional file 21 — Phylogenetic analysis of NHases. Predicted Co- and iron-containing NHase proteins are shown in green and brown, respectively. [file 1471-2164-10-78-S21.pdf]

A.

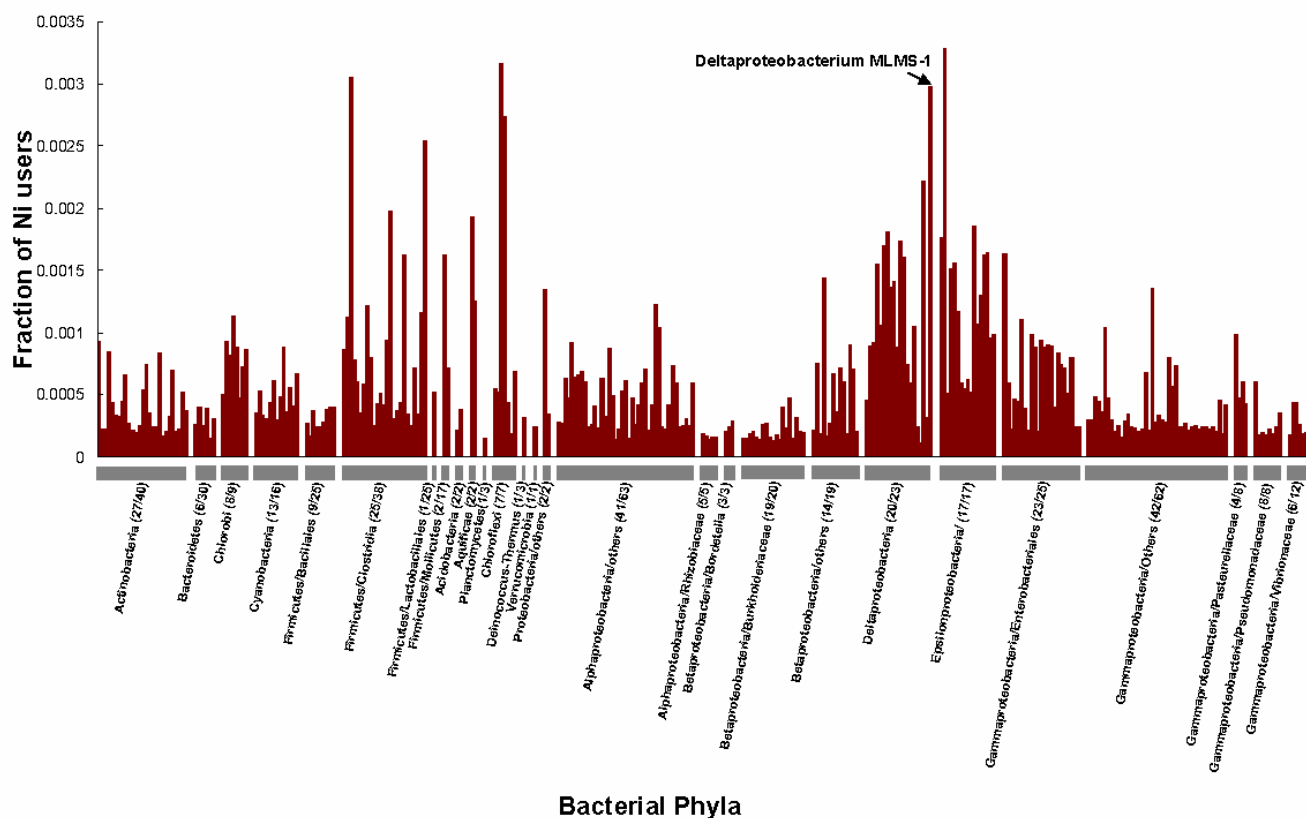

B.

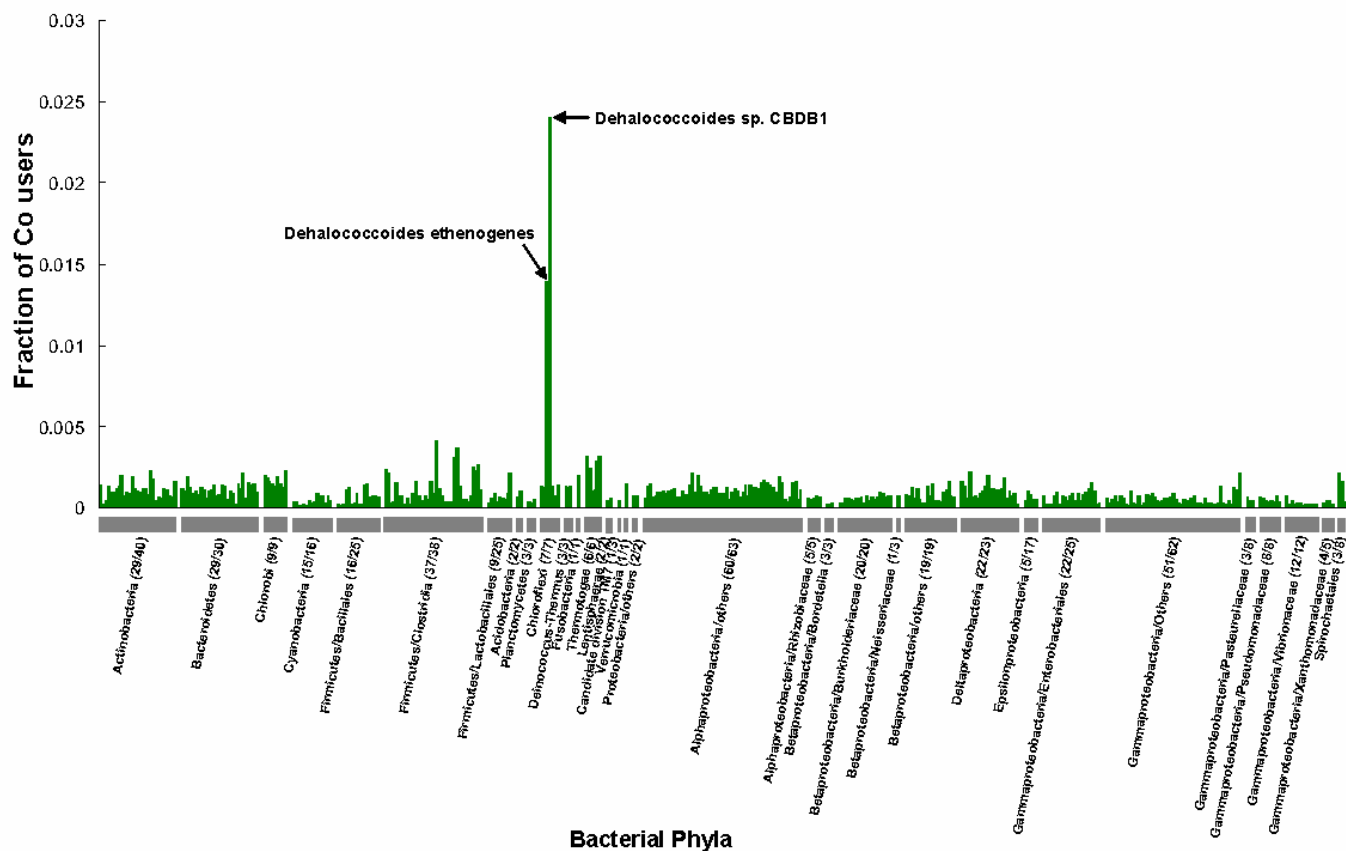

Supplement: Additional file 22 — Normalized occurrence of Ni- and Co-dependent proteins in bacteria. Each column shows a fraction of Ni- or Co-dependent proteins detected relative to the total number of annotated proteins for each organism. Numbers following the name of each phylum represent the number of organisms containing at least one user and that of total sequenced organisms, respectively. (A) Ni; (B) Co. Organisms with the largest Ni- or Co-dependent metalloproteomes are also shown. [file 1471-2164-10-78-S22.pdf]

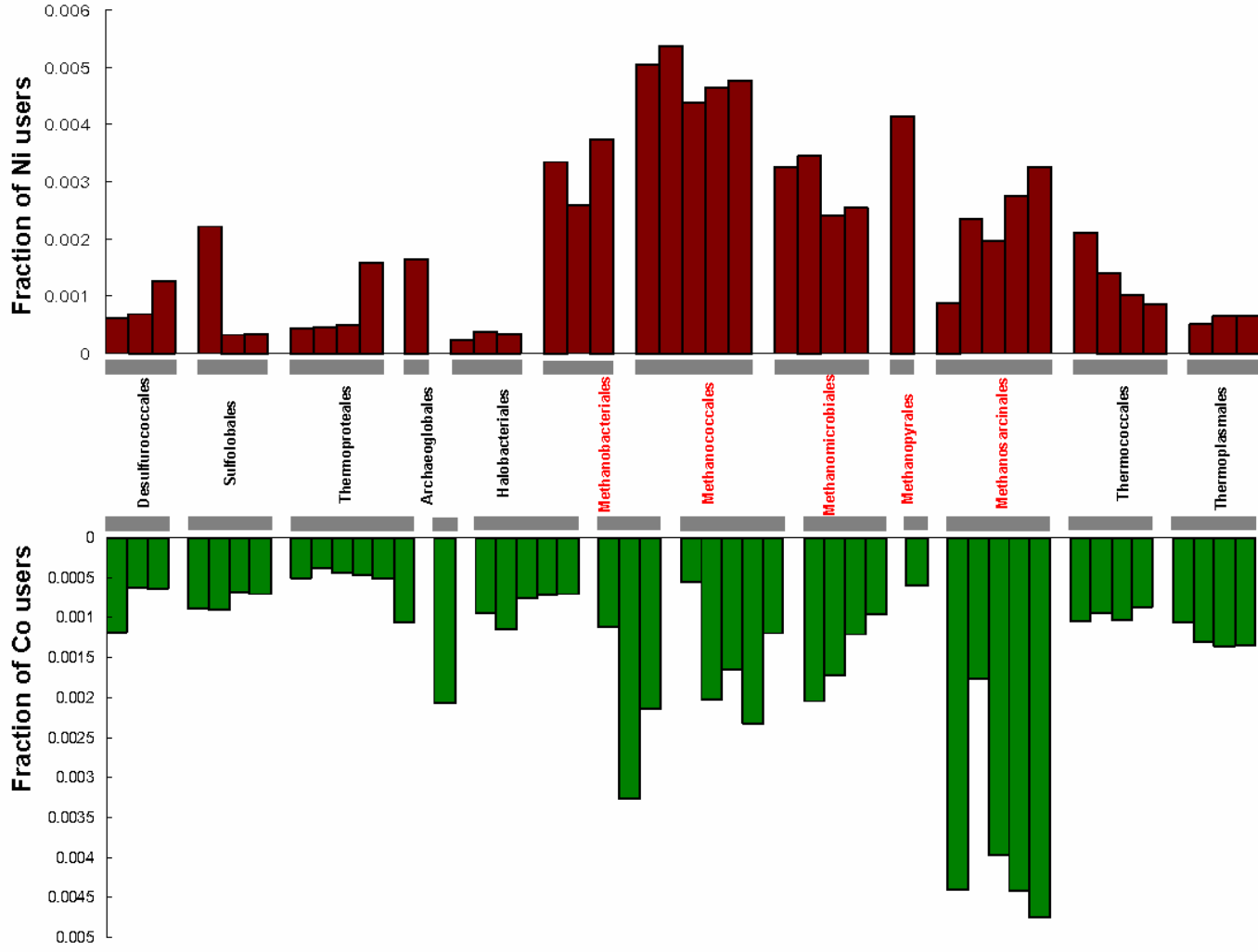

Supplement: Additional file 23 — Normalized occurrence of Ni- and Co-dependent proteins in archaea. Each column shows a fraction of Ni- or Co-dependent proteins detected relative to the total number of annotated proteins for each organism. Numbers following the name of each phylum represent the number of organisms containing at least one user and that of total sequenced organisms, respectively. [file 1471-2164-10-78-S23.pdf]

Genome size (Mbp)

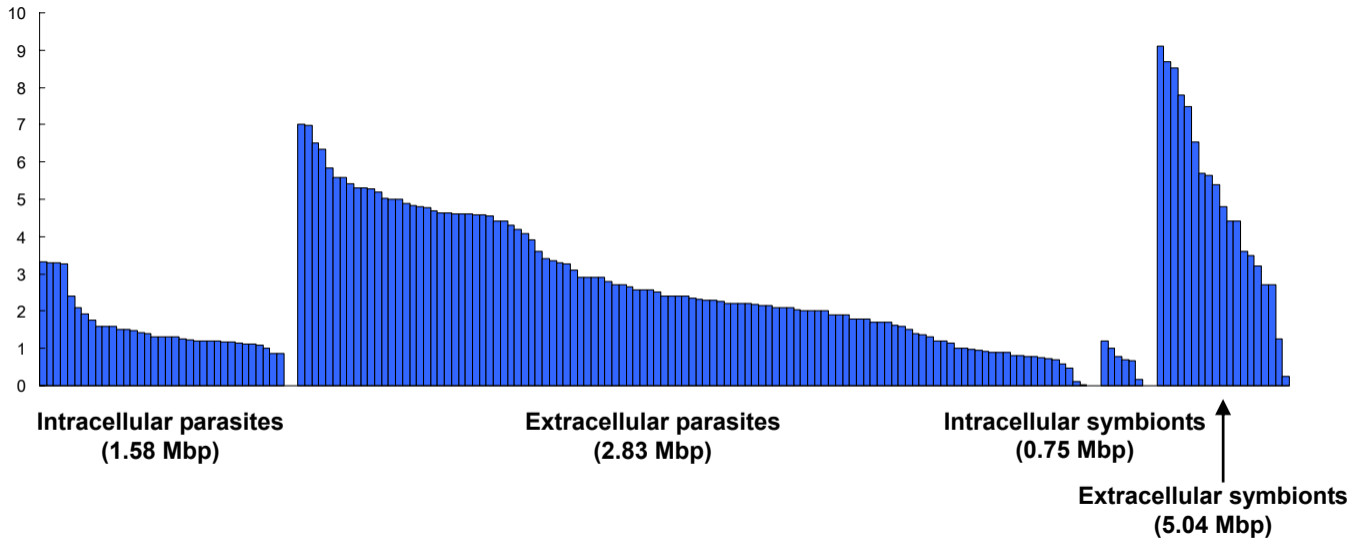

Supplement: Additional file 24 — Distribution of genome size in different host-associated organisms. All host-associated organisms were divided into four groups: intracellular symbionts (6 organisms), extracellular symbionts (19 organisms), intracellular parasites (35 organisms) and extracellular parasites (113 organisms). Each column shows the genome size of each organism in various groups. Numbers following the name of group represent the average value of genome size in each group. [file 1471-2164-10-78-S24.pdf]

**Ni utilization**

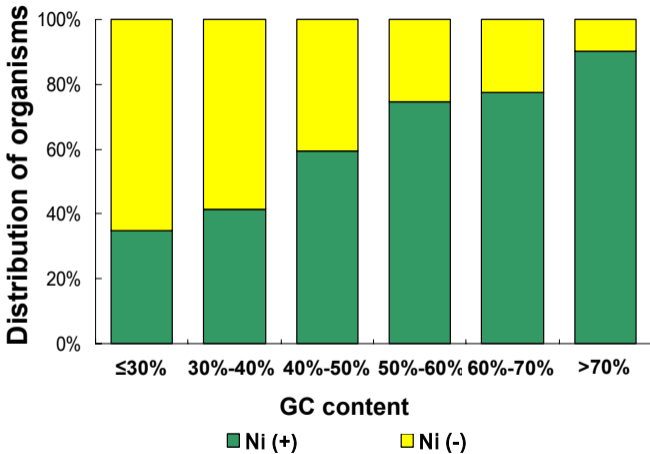

**Co utilization**

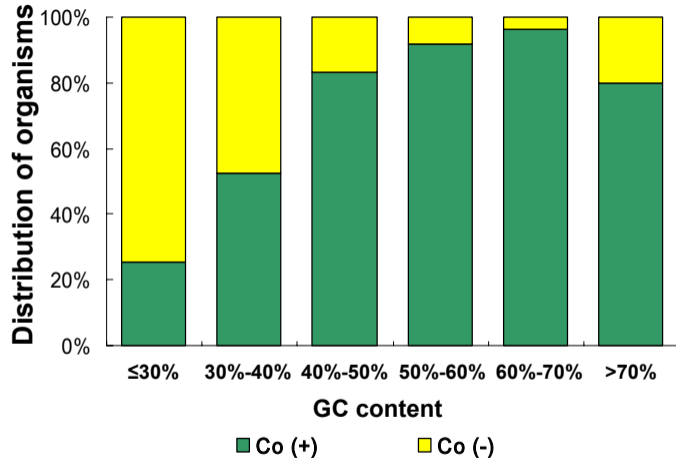

Supplement: Additional file 25 — Relationship between GC content and Ni/Co utilization traits in bacteria. All organisms were classified into four groups: Ni (+), i.e., containing Ni utilization trait; Ni (-), i.e., lacking Ni utilization trait; Co (+), i.e., containing Co utilization trait; Co (-), i.e., lacking Co utilization trait. [file 1471-2164-10-78-S25.pdf]
