# Supplementary material for: Comparative genomic analyses of nickel, cobalt and vitamin B12 utilization
Source: BMC Genomics. 2009 Feb 10;10:78. doi: 10.1186/1471-2164-10-78 (PMC2667541; doi:10.1186/1471-2164-10-78)
Supplement: Additional file 12 — Distribution of different types of NikN, NikL and NikK in bacteria. Three types of NikN, two of NikL and three of NikK were identified based on sequence similarity. [file 1471-2164-10-78-S12.pdf]

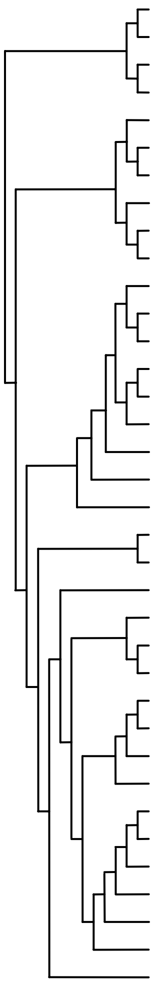

Phylum	Organisms	NikN1	NikN2	NikN3	NikL1	NikL2	NikK1	NikK2	NikK3
Firmicutes/Lactobacillales	25	-	3	-	-	-	-	-	-
Firmicutes/Mollicutes	17	-	-	-	-	-	-	-	-
Firmicutes/Bacillales	25	-	-	-	-	-	-	-	-
Firmicutes/Clostridia	38	3	9	7	-	-	-	-	-
Chlamydiae	7	-	-	-	-	-	-	-	-
Bacteroidetes	30	-	-	-	-	-	-	-	-
Chlorobi	9	-	1	5	1	-	1	-	-
Actinobacteria	40	12	-	-	-	-	-	-	-
Spirochaetes	8	-	-	-	1	-	-	-	-
Planctomycetes	3	-	-	-	-	-	-	3	-
Cyanobacteria	16	7	-	-	-	5	-	-	5
Chloroflexi	7	3	-	-	-	-	-	-	-
Deinococcus-Thermus	3	-	-	-	-	-	-	-	-
Thermotogae	6	-	-	-	-	-	-	-	-
Aquificae	2	-	-	-	-	-	-	-	-
Fusobacteria	1	-	-	-	-	-	1	-	-
Lentisphaerae	2	-	-	-	-	-	-	-	-
Verrucomicrobia	1	-	-	-	-	-	-	-	-
Candidate division TM7	3	-	-	-	-	-	-	-	-
Acidobacteria	2	1	-	-	-	-	-	-	-
Deltaproteobacteria	23	1	-	8	4	7	5	4	3
Epsilonproteobacteria	17	-	1	-	5	1	5	3	-
Alphaproteobacteria/Rickettsiales	20	-	-	-	-	-	-	-	-
Alphaproteobacteria/Others	63	1	-	-	12	1	12	8	1
Alphaproteobacteria/Rhizobiaceae	5	-	-	-	-	-	-	-	-
Betaproteobacteria/Bordetella	3	-	-	-	-	-	-	-	-
Betaproteobacteria/Burkholderiaceae	20	-	-	-	-	-	-	-	-
Betaproteobacteria/Neisseriaceae	3	-	-	-	-	-	-	2	-
Betaproteobacteria/Others	19	-	-	-	1	1	-	4	-
Gammaproteobacteria/Enterobacteriales	25	-	-	-	-	-	-	-	-
Gammaproteobacteria/Pasteurellaceae	8	-	-	-	4	-	-	3	-
Gammaproteobacteria/Vibrionaceae	12	-	-	-	8	-	-	8	-
Gammaproteobacteria/Pseudomonadaceae	8	-	-	-	-	-	-	4	-
Gammaproteobacteria/Xanthomonadaceae	5	-	-	-	-	-	-	4	-
Gammaproteobacteria/Others	62	-	-	-	6	1	4	17	-
Proteobacteria/Others	2	-	-	-	1	-	1	-	-
**Total organisms**	**540**	**28**	**14**	**20**	**43**	**16**	**29**	**60**	**9**
