# Supplementary material for: Comparative genomic analyses of nickel, cobalt and vitamin B12 utilization
Source: BMC Genomics. 2009 Feb 10;10:78. doi: 10.1186/1471-2164-10-78 (PMC2667541; doi:10.1186/1471-2164-10-78)
Supplement: Additional file 13 — Multiple alignment of a permease-like protein. This protein was only detected in six sequenced Ni-utilizing organisms. Its gene is always located within the NikMNO operon which is involved in Ni uptake. [file 1471-2164-10-78-S13.pdf]

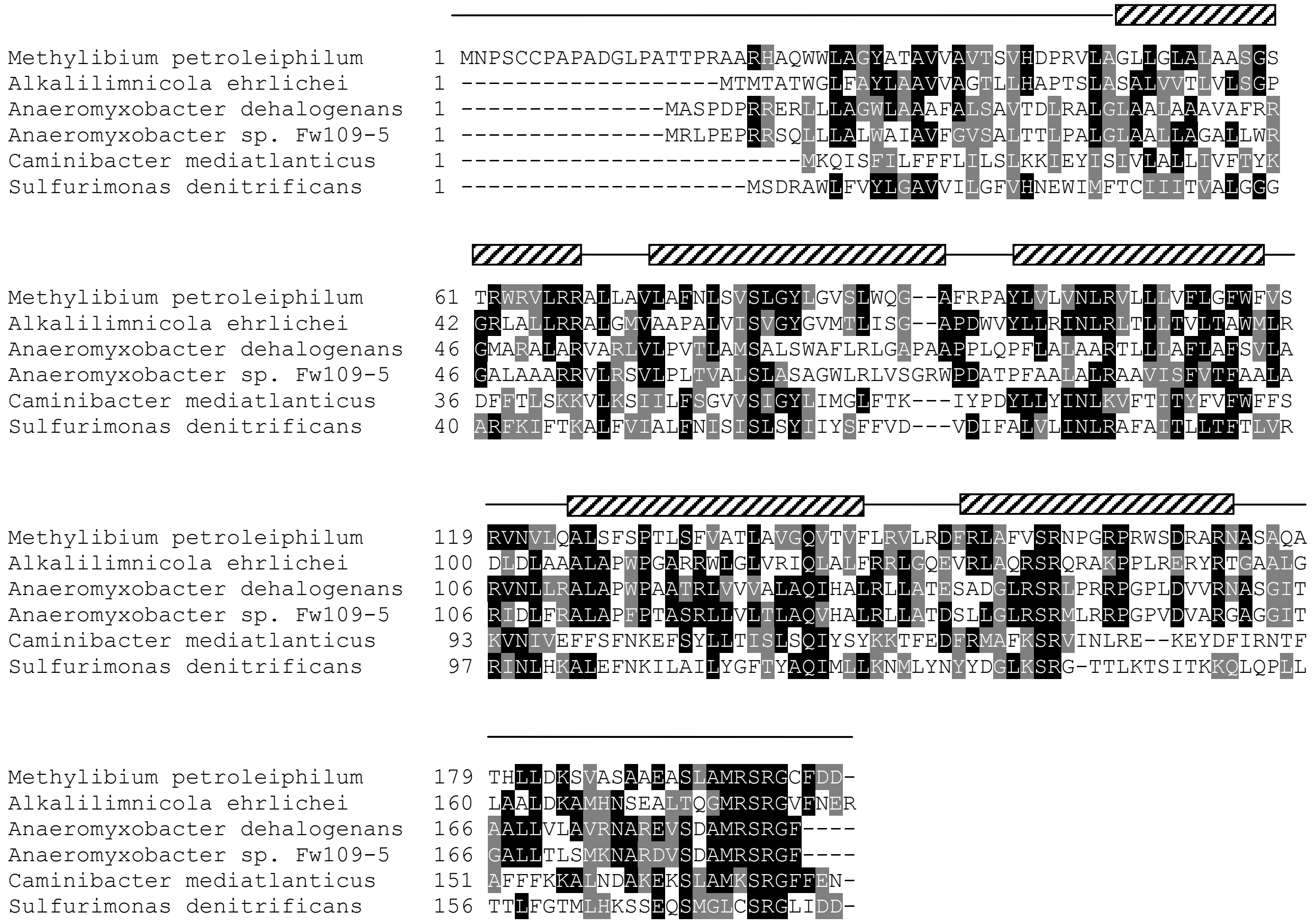

Methylibium petroleiphilum 1 MNPSCCPAPADGLPATTPRAARHAQWWLAGYATAVVAVTSVHDPRVLAGLLGLALAASGS
Alkalilimnicola ehrlichei 1 -------------------MTMTATWGLFAYLAAVVAGTLLHAPTSLASALVVTLVLSGP
Anaeromyxobacter dehalogenans 1 ---------------MASPDPRRERLLLAGWLAAAFALSAVTDLRALGLAALAAAVAFRR
Anaeromyxobacter sp. Fw109-5 1 ---------------MRLPEPRRSQLLLALWAIAVFGVSALTTLPALGLAALLAGALLWR
Caminibacter mediatlanticus 1 -------------------------MKQISFILFFFLILSLKKIEYISIVLALLIVFTYK
Sulfurimonas denitrificans 1 ---------------------MSDRAWLFVYLGAVVILGFVHNEWIMFTCIIITVALGGG

Methylibium petroleiphilum 61 TRWRVLRRALLAVLAFNLSVSLGYLGVSLWQG--AFRPAYLVLVNLRVLLLVFLGFWFVS
Alkalilimnicola ehrlichei 42 GRLALLRRALGMVAAPALVISVGYGVMTLISG--APDWVYLLRINLRLTLLTVLTAWMLR
Anaeromyxobacter dehalogenans 46 GMARALARVARLVLPVTLAMSALSWAFLRLGAPAAPPLQPFLALAARTLLLAFLAFSVLA
Anaeromyxobacter sp. Fw109-5 46 GALAAARRVLRSVLPLTVALSLASAGWLRLVSGRWPDATPFAALALRAAVISFVTFAALA
Caminibacter mediatlanticus 36 DFFTLSKKVLKSIILFSGVVSIGYLIMGLFTK---IYPDYLLYINLKVFTITYFVFWFFS
Sulfurimonas denitrificans 40 ARFKIFTKALFVIALFNISISLSYIIYSFFVD---VDIFALVLINLRAFAITLLTFTLVR

Methylibium petroleiphilum 119 RVNVLQALSFSPTLSFVATLAVGQVTVFLRVLRDFRLAFVSRNPGRPRWSDRARNASAQA
Alkalilimnicola ehrlichei 100 DLDLAAALAPWPGARRWLGLVRIQLALFRRLGQEVRLAQRSRQRAKPPLRERYRTGAALG
Anaeromyxobacter dehalogenans 106 RVNLLRALAPWPAATRLVVVALAQIHALRLLATESADGLRSRLPRRPGPLDVVRNASGIT
Anaeromyxobacter sp. Fw109-5 106 RIDLFRALAPFPTASRLLVLTLAQVHALRLLATDSLLGLRSRMLRRPGPVDVARGAGGIT
Caminibacter mediatlanticus 93 KVNIVEFFSFNKEFSYLLTISLSQIYSYKKTFEDFRMAFKSRVINLRE--KEYDFIRNTF
Sulfurimonas denitrificans 97 RINLHKALEFNKILAILYGFTYAQIMLLKNMLYNYYDGLKSRG-TTLKTSITKKQLQPLL

Methylibium petroleiphilum 179 THLLDKSVASAAEASLAMRSRGCFDD-
Alkalilimnicola ehrlichei 160 LAALDKAMHNSEALTQGMRSRGVFNER
Anaeromyxobacter dehalogenans 166 AALLVLAVRNAREVSDAMRSRGF----
Anaeromyxobacter sp. Fw109-5 166 GALLTLSMKNARDVSDAMRSRGF----
Caminibacter mediatlanticus 151 AFFFKKALNDAKEKSLAMKSRGFFEN-
Sulfurimonas denitrificans 156 TTLFGTMLHKSSEQSMGLCSRGLIDD-
