# Supplementary material for: Comparative genomic analyses of nickel, cobalt and vitamin B12 utilization
Source: BMC Genomics. 2009 Feb 10;10:78. doi: 10.1186/1471-2164-10-78 (PMC2667541; doi:10.1186/1471-2164-10-78)
Supplement: Additional file 16 — Multiple alignment of NikA and other homologs. Residue sets proposed to be involved in Ni-binding in E. coli by various groups are shown in different colors. Ligands suggested by Cherrier et al. are highlighted in red background and those suggested by Addy et al. in blue background. Other residues shown in white on black or grey are conserved in homologs. [file 1471-2164-10-78-S16.pdf]

```
NikA Escherichia coli                 1 ------------MLSTLRRTLFALLACASFIVHAAAPD-------EITTAWPVNVGPLN-PHLYTPNQMFAQS-------MVYEPLVKYQADGSVIPWLA
NikA Shigella boydii                  1 ------------MLSTLRRTLFALLACASFIVHAAAPD-------EITTAWPVNVGPLN-PHLYTPNQMFAQS-------MVYEPLVKYQADGSVIPWLA
NikA Brucella suis                    1 ----------MLTDRLRGLMLAFAFVSVAFVSGAHADP-------KLNFSWPVNVGPLN-PHLYSPNQMFAQN-------MVYEPLVHYNADGTVGPWLA
NikA Alkaliphilus metalliredigens     1 -----MKCNKIVVMILVLALSFSMVGCNTATTQASGDIGEE--KTQLVYASTKDIRDIN-PHTYSG-EMAAQN-------MVFESLVINTDEG-VKPWLA
NikA Bacillus halodurans              1 ---MCTLNRKLILLFVISLISSILVGCAESESGTVSNEGEENTEKSITFSWPRDIGPMN-PHVYNPSQLFAQS-------MIYEPLVSYTEGGELQPHLA
NikA Helicobacter hepaticus           1 ----------MRAIVIVFICMGVLWG-----------------KNTLIMAVSENIGALN-PQGYQGNAMFAQN-------AIYEGLVRVDKQGKIVPSLA
YntA Shewanella pealeana              1 -------------MKSLILFILSIS--------LLPLPSVASTDKVLTLA--LGAEPETGFDPIYGWGKYNRP-------LFQSTLIKRDNALALVSDLA
YntA Methanosarcina mazei             1 -MSSPAADSKPTEQTSQVAGETALQ--------GSEELSSPGSDELVAAVGTHGGEPETGFDPISGWGYNHEP-------LVQSTLFKKDSEANLINDLA
YntA Salmonella typhimurium           1 MLRREKQKQRIIWDKLMIKGKLALVTCALTLVFTSSLFAASDTADGRTLKLAIGPEPTEGFDPMLGWSHGSYL-------LLHAPLLKQNADMSWGNLLT
Co Desulfitobacterium hafniense       1 ---MKVKMKKWLAVALSGVLVLALAACSQDVSKQGGSPASDSGEQTVHLNLPESWGFESFYPVLTPANSSSGYGITYYLTSFYDTLVTYDDKGELAGLLA
Co Clostridium tetani                 1 ------MLKRSFNKKNIFNKLLALVMVSSIALSGCGTKSEKDTTKTDSNKEIVLASPRDVAPGKENVHFANVLS------YVWEPLVSMNKKGEPTPKLA
DdpA/OppA Pseudomonas syringae        1 -------MKPLVRSLLASALVLAAG------------------SVSAQDLRIGYADPVSSLDPQLNNYAGDRS-----VALHAFESLVSRRDDKTLPGLA
DdpA/OppA Hahella chejuensis          1 -------MGRLSGVLRVLVCCLSFFALTSPSAAQTPAN--------VLVVGQIAEPKSLDPHAVTALNDFRILIN-----LYDGLVRYKDGTLEVEPALA

NikA Escherichia coli                74 KSWTHSEDGKTWTFTLRDDVKFSNGEPFDAEAAAENFRAVLDNRQRH------AWLELANQIVDVKALSKTELQITLKSAYYPFLQELALPRPFRFIAPS
NikA Shigella boydii                 74 KSWTHSEDGKTWTFTLRDDVKLSNGEPFDAEAAAENFRAVLDNRQRH------AWLELANQIVDVKALSKTELQITLKSAYYPFLQELALPRPFRFIAPS
NikA Brucella suis                   76 ESWEASQDGRSYTFKLREDVKFSNGEVFDAAAVKANIDTVLQNRPRH------NWLELVNQMVSAEVVGPYKVRINLKKPYYPLLQELSLPRPFRFIAPS
NikA Alkaliphilus metalliredigens    84 ENWEISEDGKTYTFKLREDVKFTDGEVFNAAAAKMNIDAVMNNFERH------AWLELVNQIDHVDIIDDFTIDLVLKNAYYPTLSELALTRPFRFISPN
NikA Bacillus halodurans             90 DSWTISEDGKEYTFKLREGVQFSDGTPFNAEIVKKNFDTWIEHSSLH------SWLGVMNVLEKTEVVDEFTFKMVLKEPYYPALQDLAVVRPVRFLGEA
NikA Helicobacter hepaticus          66 LSWNTSQDGLSYEFVLRKGVKFSNGEAFNADAVVLNFQSILKNRARH------SWSGLAMAIKDMQKVSEYKVRLLLKHPYSPTLNELAVVRPFRFLAPS
YntA Shewanella pealeana             71 LRWRLSDDRLVWSVDLRQGVKFNDGTGLTASDVKFSFDTAKQS----------ASLHDLTQLERVEIKGDYSVEFYLKQADITFIDNLVNLA----IVPK
YntA Methanosarcina mazei            85 TDYTISDDGLKWTVKIRSDVKFHDGEPLTAKDVAFTFNTAADA----------SGSVDLSVLEKATAVDDYTVEFNLNDPQSTFINKLVALG----IVPE
YntA Salmonella typhimurium          94 EKVDTSPDGKIWTLTLKPGLKFSDGSPLTAEDVVFTYNKAAKS----------GGKIDMGNFSHARALDARRIEMTLSHPQSTFVNVLGSLG----IVPA
Co Desulfitobacterium hafniense      98 EEWSVSPDGKVYTFKLREGVKFSDGTPLTAEDAAQSLLAVPVNLGQYNG----GYGKLSTIIADAVAKDDYTLELHLTQPYYSTLRDLCLANPFAIVSSE
Co Clostridium tetani                89 KSWEMSKDAKEWTFKLQEGVTFHNGEKFNADIVVANFDRYKKLKNGKSNFYTFKVDKIYPGLIDCVKTDDYTVKLNFKKPIPTLAYNMVNFGSPIFHPND
DdpA/OppA Pseudomonas syringae       71 KSWKVTDDT-TWEFALREDVKWQDGTPLTADDLVFSFERARSVPG--------SVASYAGAMRTVESVKAKDDHTLIIKTRLPNANLLPDVDSIYIVSRH
DdpA/OppA Hahella chejuensis         81 ESWTISDDGKIYTFTLRKGVLFHDGAPLTAEAVKFNFERMLDENHPYHDTGPFPLAFFFSPIEKVEAIDDLQIRFVLKAPYAPFLSNLAYPTGLIISPDS

NikA Escherichia coli               168 QFKN-HETMNGIK-APIGTGPWVLQESKLNQYDVFVRNENYWGEKPA----IKKITFNVIPDPTTRAVAFETGDIDLLYGNEG--------LLPLDTFAR
NikA Shigella boydii                168 QFKN-HETMNGIK-APIGTGPWILQESKLNQYDVFVRNENYWGEKPA----IKKITFNVIPDPTTRAVAFETGDIDLLYGNEG--------LLPLDTFAR
NikA Brucella suis                  170 QFKN-GGTADGIV-APIGTGPWKLTETKLGEHDVFTRNDSYWGPKPA----YEQITVKVIPDPNTRAIAFEAGEIDLIYGTEG--------PISPDTFER
NikA Alkaliphilus metalliredigens   178 SFID-GNTKEGVN-GYIGTGPWVLSEHKENQYAIFTANESYWGEKAK----LNAVKWQVMPDHQTILLALEKGEIDLLFGADGD-------MVDLDSFKA
NikA Bacillus halodurans            184 GFPDDGDTSQGIK-EPIGTGPWMLSDYKQDEYAVFTRNPNYWGESPK----IDKVTVKIIPDAETRVLAFESGELDLIFGEGV---------ISMDAFNQ
NikA Helicobacter hepaticus         160 AMPKDLDLLQHNP-KPIGTGAYMLGKSKLGVSDTLLKNPHYWNQDAYNGIYYDEIILKVIFDPNAKLAALKSKQIDMIYGYDQ---------IPLEIFKN
YntA Shewanella pealeana            157 ASYG-AQYGL----KPIGSGPLAFVRWDRNQQLIMEPNPYYYGQKPE----FSKVIVTFS-DEDSRYARLRSGQLDLAAIAPRYAKILPPG-YKLWSIDS
YntA Methanosarcina mazei           171 HAYNSETYGS----NPAGSGPYKFVQWDKGQQVIFEANPDYYGQEPY----FKKLTILFM-DSDAAFAAAKSGQIDLAEVPSTFADQKVDG-MKIVSLES
YntA Salmonella typhimurium         180 SRYDEKTFAR----EPIGAGPYRLVSFQPGQQLIVEANPWYAGKKND----FNRLVFVFL-DEDNAYAAARSGQLGLVRIAPSMAVAPQQDNLKLWVRDS
Co Desulfitobacterium hafniense     194 QLNGDLTAKDSFTTVSYGTGPYMYGGDGDGKKYTFVRNPHYWGEQPD----VDSFTIKVIADNDAKVLALKNGELDFMSGIAKVS--------SESYQEM
Co Clostridium tetani               189 FGKDGNFIVD----KPNATGPFKLIESKKNSYCLLEAFDNYWGEKAK----SKKIRIKTIPEGQTRYSALKAGEVMGVLDLGA---------ITPNLAEE
DdpA/OppA Pseudomonas syringae      162 AGAAASSADYNSGKALIGTGPYRFVSFVPGDRTIFARNDSYWGAKPT----WDKVDFRFIANAANRTAALLAGDVDVIDKVSPTDVERLRKTPSVNVFAY
DdpA/OppA Hahella chejuensis        181 VKKYAKDYGR----HPAGTGPFRFAEWVSNAKVVIVRNENYWDDAPP----LEAVVFRPITDANTRMAEMLSGGVDLMVETPP------------DNLAV

NikA Escherichia coli               254 FSQNPAYHTQLSQPIETVMLAXNTAKAPTNE------LAVREALNYAVNKKSLIDNALYGTQQVADTLFAPSVPYAN-LGLKPRQYDPQKAKALLEKAGW
NikA Shigella boydii                254 FSQNPAYHTQLSQPIETVMLALNTAKTPTNE------LAVREALNYAVNKKSLIDNALYGTQQVADTLFAPSVPYAN-LGLKPRQYDPQKAKALLEKAGW
NikA Brucella suis                  256 FQKMGIYNTELSEPLETRVLALNTNHGATKD------LAVRKAINHAVDKDTMIATVLYGTQKRADTLFADNVPYAN-IGLKPYAFDPALAARLLDEAGW
NikA Alkaliphilus metalliredigens   265 LEDRGKYVTVMSEPIASRAILLNSNKEITGD------IKVREALQYAIDKEGIAEGILNGSESVADTVLSPTVAYCN-VDLEKRSYDIDKASKLLDAAGW
NikA Bacillus halodurans 1          270 LKESGQYGTDLSEPVGTRSLLLNTSNEKLAD------LRVRLALHHGFNKQAMVEGVTLGLEEKADNILSTNFPYTD-IDVEPIEYDVEQANAYLDEAGW
NikA Helicobacter hepaticus         250 MQNDKHFNTYLSPPIYSTILVINSASPILTLSNEKFSQNLRKAIALGIDKTKITQAVYGGLQEKADTIFAPNIFHSL-SSYDVLKILPYKPKEAQNAIVL
YntA Shewanella pealeana            246 VDNRGIAWPMVATQADN------IGNDVTSD------AAIRQAVDMAVDRDVLVQQLLDGFATPAYSIADG-LPWGP-AQTQSSQPDLAAAKRLLEQNGW
YntA Methanosarcina mazei           261 IDARGISFPMNPNTGEKTENGYVIGNDVTSD------PAIRKALNIGIDRQALVDGALNGQGEEEFTGVDK-LPWGN-KEAIFEDGDIEGAKKILAEGGW
YntA Salmonella typhimurium         271 VENRGIVFPMVP-AGKKDANDYPVGNDVTAD------VAIRRAINYAINRKQLAEQVMEGHAIPAYSAVQG-LPWQN-PSVIFSDGDIAKARAILEEAGW
Co Desulfitobacterium hafniense     282 KNTKGFGAAVDKAPTQTYYLGYNLKNPLFAE------QVVREAMAAAIDKGNIVEGIFGGIHEQADTFFAQSLPYCD-VEQTTYEFDLAKANRLLDDAGY
Co Clostridium tetani               272 LLKDSKFESSIEKSTINHFLSLNGTKFPFND------PKMKEAVSLAIDRNLIVDKFYKGFGNPTQNLLNHVSPFYK---EMKPEYNLEKAKKLASEVLK
DdpA/OppA Pseudomonas syringae      258 QGLRALIIQPSFRTGPNEFIRDNAGKPLAEN--PLLDVRVRKALSLAINRPAIDERIMQGTVTEANQWMPANTFGYN-PGIKNIPYDVKQAKDLLAQAGF
DdpA/OppA Hahella chejuensis        261 FEKDPGFQVYQQAGPHLWFLILNNKKGPFAD------RRMRQAINYAIDKKALVENVLQGTAEIAKGPIPPAFAWAYNTNLDTYAYNPDKARALIKAAGR

NikA Escherichia coli               347 TLPAGKDIREKNGQPLHIELSFIGTDALSKSMAEIIQADMRQIGADVSLIGEEESSIYARQ---RDGRFGMIFHRTWGAPYDPHAFLSSMRVPSHADFQA
NikA Shigella boydii                347 TLPAGKDIREKNGQPLRIELSFIGTDALSKSMAEIIQADMRQIGADVSLIGEEESSIYARQ---RDGRFGMIFHRTWGAPYDPHAFLSSMRVPSHADFQA
NikA Brucella suis                  349 TAKASGDIREKDGQPLAIELCFIGTDAISKSMAEIVQADLRKVGIDVKLTGEEESSIYARQ---RDGRFDMIFNQTWGAPYDPHAFVSSMRVPSHADYQA
NikA Alkaliphilus metalliredigens   358 TLGN-DGYRYKDGAKMEITIYYNSDNSQERMISEYMQDNLKNAGVNLKIVGEEKQAFLDRQ---KTGDFDLQYSLSWGIPYDPQSYVSSWRIPAHGDYQA
NikA Bacillus halodurans            363 ELPAGKTVREKNGEQLELELIYDKTDPLQKAMAETMQAEWAAIGVKLDITGLELTTQIQRR---RAGDFDVDFWYNYGAPYDPHSFINVVAEAGWGVAEA
NikA Helicobacter hepaticus         349 LLQDSQAALLRD---RGVEILFSGDNPAHKMIAEILQSEFKAIGIKARLSASEPTIYRNRL---LKGAFDIAFSETWGAPYEPLSILYSMLIPSHIDFAA
YntA Shewanella pealeana            332 QVKQGR--RYRDGVAASMTLYYLAGDTVRQQLALAVAQMVKPLGIEIKPEGKSWEAIYLQM---HRQPVLLGFGSHSATEVRAVYHSASRGKGYFN-SGY
YntA Methanosarcina mazei           353 TDTDGDGIVEKSDLKAEFTLLYPANAQERQALAVSMGEEAKKLGINIKVEGKSWDEIYTLC---YSTPNVWGYGSLDPTDIYLRYYSKSYDPSTRNNVIM
YntA Salmonella typhimurium         362 KINSAG-VREKAGKEARLTLWYASGDSTRRDLAEAVRAMLQPLGIVVSLQSGSWETVERHM---HANPTLFGWGSLDPMELFHHYSGKAAGVEYYN-PGY
Co Desulfitobacterium hafniense     375 VDRDGDGIREINGTRLAAEFVYQTESVANNDMVVYLCDQFKKIGVELTPKSAPMMEWYAMI---TGGQYGVTVFKTQGGYYDPANIVSAMNPSQSMDPII
Co Clostridium tetani               363 ---------GKNTNVTMIIPSEFTKKYPYKEEAQYIQSVLKELGINVNINIYDFATFNQIR---NKGDFNLQMHIQGLPNMEPYTMFDNYMRSNGSTNKN
DdpA/OppA Pseudomonas syringae      355 PEG-----------FQLTVHVPGDRYPQAPEVMQAVAQFWTRIGVKVQLEVLPWAVYAGKANKNELAISVIAWGNGTGEAAYALTNILTTVDSSKGQGAS
DdpA/OppA Hahella chejuensis        355 EGAQLTFYVTEGGSGMLEPVAIG----------AAIQADLAKVGLQVKIVTYEWNTFLSKVNPGLPGDVDMAEMAWMTNDPDTLPYLALRSDAWPDKGGF

NikA Escherichia coli               444 QQGLADKPLIDKEIGEVLATHDEAQRQALYRDILT--RLHDEAVYLPISYISMMVVSKPELGNIPYAPIATEIPFEQIKPVKP--------
NikA Shigella boydii                444 QQGLADKPLIDKEIGEVLATHDETQRQALYRDILT--RLHDEAVYLPISYISMMVVSKPELGNIPYAPIATEIPFEQIKPVKP--------
NikA Brucella suis                  446 QLGLPDKAKIDAEIGQVLVSTDETARQALYKDILT--RLHEEAVYLPLTSVTAMAVAKPEVGKITFGAMSSEIPFEKLTPKAN--------
NikA Alkaliphilus metalliredigens   454 QVGLEKKAWLDEMIGNVMIETDENTRQEMYNEILT--YIHDASVYIPLTYSRTKAVYEPSLRGVGFNTSQYEIPFENMYFQQ---------
NikA Bacillus halodurans            460 HSNLSMKEELDEQVRATLASTDETERQELYGSIIN--TLQEQSVFVPISYIKKTVVYQENVNEFIFPANRDEHPFNGIDVSN---------
NikA Helicobacter hepaticus         443 QAGLSQKPHIDKLIREIIALNPHS-KVFYSALSEVIALLQESGVYIPLTYQRNKVIAHKKIKGIKMGVVGYEVPFWEMYE-----------
YntA Shewanella pealeana            426 YSNPEVDRALDSAQAAASWSESLPYWQQAQAL------IAADRPWTWLVNLKHLYAANECLDLAKPGIEAHGHGWPLANNIEQWRWQCPSR
YntA Methanosarcina mazei           450 YNNPAVDNYLRTAITSPDQEAANKNWQLAAWDGTTGFSAEGDASWMWMATINYVYIMDEDLDIGTPKIQPH--GADIFGSILEWKRA----
YntA Salmonella typhimurium         457 YSNPAVEAHLKQAIDAPDWQKAIPFWQQVEWDGKQGAGVQGDAAWAWLLNIQHTYLANPCIDLGKGAPEIHG-SWSVLNNLDDWTWTCR--
Co Desulfitobacterium hafniense     472 MQAGGFLPDGVNLINEVNSTTDEARIEEIYATIIN--TIADNCLMTPLYYTHQVALYNDKVAGYDFPGDASFTSIQNIKAAK---------
Co Clostridium tetani               451 YHFGYKNDKVDNLMNKLDETLDIEKRAAIYDELQS--ISAKDPSTIPLFSEMNLIVSNKEVTGHNAFIYGTTLPELQWSK-----------
DdpA/OppA Pseudomonas syringae      444 NWGHYSNPLVDKALADSTAEFDEAKRRKILEDSVQ--VVSDDVGIIPLFHYQNIWDARKGLKVEPLVSDRTAATMVTEQP-----------
DdpA/OppA Hahella chejuensis        445 NSGYYANPKVDDLLERARRATDQDERATLYKEMQF--IVEEDAPWAFIANWKQNAVSNTRVKNFKLQPSFFLDLTAVNKL-----------
```
